# Characterization of age signatures of DNA methylation in normal and cancer tissues from multiple studies

**DOI:** 10.1186/1471-2164-15-997

**Published:** 2014-11-19

**Authors:** Jihyun Kim, Kyung Kim, Hyosil Kim, Gyesoon Yoon, KiYoung Lee

**Affiliations:** Department of Biomedical Informatics, Ajou University School of Medicine, Suwon, 443-380 South Korea; Department of Biomedical Sciences, The Graduate School, Ajou University, Suwon, 443-380 South Korea; Department of Biochemistry, Ajou University School of Medicine, Suwon, 443-721 South Korea

**Keywords:** DNA methylation, Epigenetics, Age signature, Aging, Meta-analysis, Systems biology, Cancer

## Abstract

**Background:**

DNA methylation (DNAm) levels can be used to predict the chronological age of tissues; however, the characteristics of DNAm age signatures in normal and cancer tissues are not well studied using multiple studies.

**Results:**

We studied approximately 4000 normal and cancer samples with multiple tissue types from diverse studies, and using linear and nonlinear regression models identified reliable tissue type-invariant DNAm age signatures. A normal signature comprising 127 CpG loci was highly enriched on the X chromosome. Age-hypermethylated loci were enriched for guanine–and-cytosine-rich regions in CpG islands (CGIs), whereas age-hypomethylated loci were enriched for adenine–and-thymine-rich regions in non-CGIs. However, the cancer signature comprised only 26 age-hypomethylated loci, none on the X chromosome, and with no overlap with the normal signature. Genes related to the normal signature were enriched for aging-related gene ontology terms including metabolic processes, immune system processes, and cell proliferation. The related gene products of the normal signature had more than the average number of interacting partners in a protein interaction network and had a tendency not to interact directly with each other. The genomic sequences of the normal signature were well conserved and the age-associated DNAm levels could satisfactorily predict the chronological ages of tissues regardless of tissue type. Interestingly, the age-associated DNAm increases or decreases of the normal signature were aberrantly accelerated in cancer samples.

**Conclusion:**

These tissue type-invariant DNAm age signatures in normal and cancer can be used to address important questions in developmental biology and cancer research.

**Electronic supplementary material:**

The online version of this article (doi:10.1186/1471-2164-15-997) contains supplementary material, which is available to authorized users.

## Background

DNA methylation (DNAm) is one type of epigenetic modification that regulates gene expression heritably. It is catalyzed by DNA methyltransferase that adds and maintains a methyl group to the 5′ position of the cytosine ring to form 5′ methyl-cytosine [1]. In mammalian genomes, this modification occurs almost exclusively on cytosine residues that precede guanine (CpG dinucleotides). These CpG dinucleotides are generally about 60%–90% methylated [2]. However, CpG islands (CGIs), which are the genomic regions with the highest CpG density, exhibit the lowest levels of DNAm [3]. The potential role of aberrant DNAm in human diseases such as cancer, both at a single-gene level and on a genome-wide scale [4], is important.

Recently, DNAm has also been shown to be associated with aging in a wide array of organisms, ranging from yeast to humans [2, 5, 6]. For example, Horvath *et al.* observed that a genome-wide decrease in DNA methylation, preferentially hypermethylation at CGIs, occurred during aging [7]. Some studies have investigated age-associated methylation of CpG loci dependent on sex, body mass index, specific tissue, or cell type [8–12]. These studies were performed using various linear-based methods, including conventional linear regression methods [11, 12], a weighted correlation network method [8, 10], and a multidimensional scaling method [9]. However, most of these studies were restricted to specific tissue types, and included limited numbers of samples and/or limited ranges of sample ages. More recently, several studies were performed to identify more reliable CpG sites associated with aging, which collected many samples with various tissue types from public data sets [13–16]. These studies also used linear-based regression methods of analysis. Although previous studies have reported that age-associated DNAm can show both nonlinear and linear patterns [12], there has been little identification of age-associated DNAm signatures through systematic analysis of nonlinear DNAm patterns. More importantly, the characteristics of DNAm age signatures that are applicable to multiple types of normal or cancer tissues are not well studied.

In this study, we identify for the first time tissue type-invariant DNAm age signatures for healthy normal and cancer tissues using linear and nonlinear models. For more reliable signatures, we collected diverse samples from a range of studies available in public resources that included multiple tissue types. After identifying the DNAm age signatures from normalized DNAm levels of the samples, we extensively investigated the characteristics of the signatures and their biological meaning through a number of analyses, including analysis of changes in DNAm pattern with age, gene ontology term analysis, and network and conservation analysis. We also compared the signatures with the results of previous studies. Finally, we checked that the signatures could be used as an age predictor for multiple tissue types.

## Results and discussion

### Discovery of age-associated DNA methylation signatures

**Figure 1:**
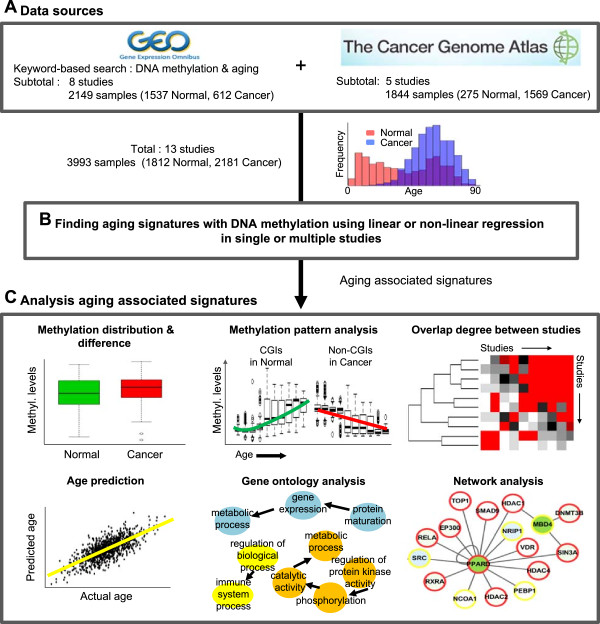
**Study overview. (A)** Sources of DNAm data with sample information. Eight studies from GEO and five from open TCGA data were included. **(B)** Identifying an age-associated DNAm signature. Linear and nonlinear regression models using single or combined studies were applied. **(C)** Age prediction and characterization of identified age-associated signatures. Various analyses using DNAm patterns and distributions, gene ontology, and protein networks in normal and cancer tissues were performed.

**Figure 2:**
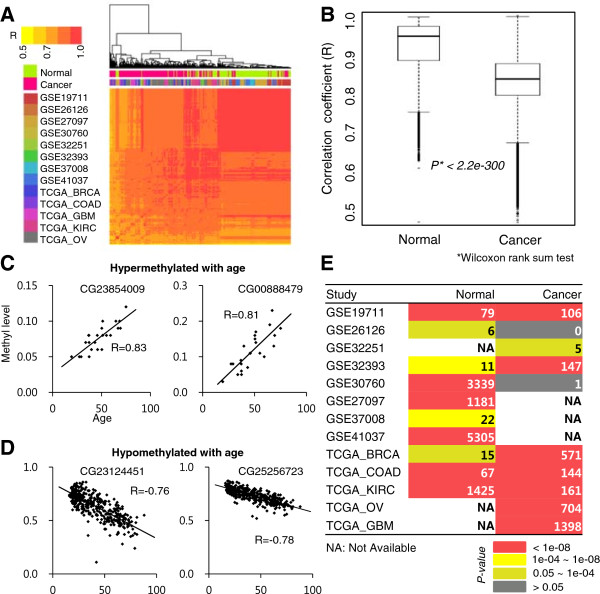
**DNA methylation correlation and age-associated CpG loci across individual studies. (A)** Heat map representing the Pearson’s correlation coefficients between all samples of normal and cancer tissues used in this study. A hierarchical clustering was used. **(B)** Box plot representing the correlations between normal samples or between cancer samples. *P*-value was calculated using a Wilcoxon rank-sum test. **(C)** Examples of CpG loci (i.e., CG23854009 and CG0888479) hypermethylated with age in the GSE32393 study. **(D)** Examples of CpG loci (i.e., CG23124451 and CG25256723) hypomethylated with age in the GSE41037 study. R: correlation coefficient. **(E)** The number of significant age-associated CpG loci in normal or cancer tissues from individual studies. The number and color in each cell corresponds to the number of significant age-associated CpG loci and its significance in terms of a *P*-value, respectively. *P*-value: Z-test result using the random distribution of the 100 age-permutation tests. NA: not available.

To identify robust age-associated DNAm signatures, we first searched and downloaded various DNAm profiles from diverse studies available in the Gene Expression Omnibus (GEO) database (http://www.ncbi.nlm.nih.gov/geo/; Figure 1). We then excluded studies without age information or with small numbers of samples (<10). We also excluded samples of diseased tissues other than cancer. It is known that technical bias exists across different array platforms [17], so we considered only the Illumina Infinium HumanMethylation27 Bead Chip array, which was the most widely used among the downloaded profiles. Consequently, we collected DNAm profiles of 2149 samples (1537 disease-free normal and 612 cancer samples) from eight studies available in the GEO. Additionally, we downloaded 1844 publicly available DNAm profiles (275 normal and 1569 cancer samples) of five cancer types (breast, ovarian, glioblastoma, kidney, and colon) evaluated on the same platform from The Cancer Genome Atlas (TCGA) consortium [18–22]. In total, we gathered DNAm profiles of 1812 normal healthy and 2181 cancer samples. These samples included diverse tissue types and exhibited a wide range of ages from 0 to 91 years (Additional file 1: Table S1). We next normalized DNAm levels (range from 0 to 1) using a single beta-score measure that indicates conceptually the normalized levels of DNAm (Methods). The normalized DNAm levels were well correlated between normal or cancer samples, but had higher correlation scores between normal samples (Figure 2A,B), and for both normal and cancer tissues, the DNAm levels in CGI regions of individual samples were much lower than those in non-CGIs (Additional file 2: Figure S1). Moreover, the DNAm levels of normal and cancer tissue showed different patterns depending on the genomic regions. In CGIs, for example, the average DNAm levels were generally higher in cancer than those in normal tissue, except for ovarian cancer samples (Additional file 2: Figure S1).

Next, using various regression methods, we identified an aging signature associated with the normalized DNAm levels. Because some studies have reported that methylation levels can change most dramatically during childhood [12], we applied nonlinear regression models as well as linear regression models. After finding statistically significant age-associated DNAm sites using single studies or combined multiple studies, we examined the characteristics and biological meaning of the sites through analysis of the associations of DNAm patterns with age, gene ontology, sequence conservation, and protein networks. We also compared the age-associated DNA loci across different studies or tissue types. To identify tissue type-invariant age-associated DNAm signatures, we integrated data from all samples of normal tissues, cancers, or both, after removing noisy samples (Methods). We compared the methylation patterns of the normal signature to those in cancer according to different genomic regions. We finally checked the potential for age prediction using the methylation levels of the age-associated signature, regardless of tissue type.

### CpG loci are widely associated with age in disease-free normal samples

The distribution of DNAm levels showed a significant disparity between normal and cancer tissues depending on the genomic region. We first checked age-associated DNAm sites separately in normal or cancer samples using a linear regression model. For example, CG23854009 (located at 62802940 on chr19; correlation coefficient R = 0.83) and CG00888479 (19141824 on chr20; R = 0.81) sites showed linear hypermethylation patterns according to age in normal samples from the GSE32393 study [23] (Figure 2C). In contrast, hypomethylation patterns were observed at CG23124451 (37878077 on chr20; R = -0.76) and CG25256723 (167822568 on chr1; R = -0.78) in normal samples from the GSE41037 study [7] (Figure 2D). We checked the number of significantly age-associated CpG loci in each study (*P* < 0.0001 by linear regression). The numbers of significant CpG loci were quite different between studies (Figure 2E), mainly because of different ranges of age and/or different numbers of samples across studies (Additional file 2: Figure S2). Although the numbers of age-associated loci in normal tissues varied between studies, the numbers were significant in all studies included (*P* < 0.05 using a Z test of 100 age-permutation tests; Figure 2E). In cancer samples, however, the numbers of age-associated loci were not significant in some studies, including GSE26126 and GSE30760.

**Figure 3:**
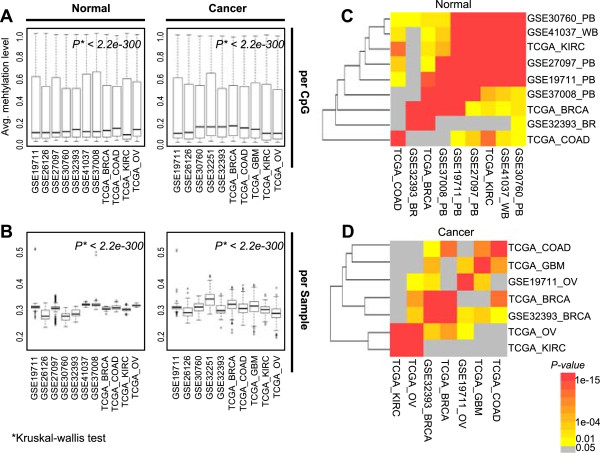
**Comparison of age-associated CpG loci across different studies with different tissue types. (A)** Box plots of average methylation values (y-axis) per CpG unit in normal and cancer tissues across individual studies (x-axis). **(B)** Box plots of average methylation values per sample unit in normal or cancer tissue across individual studies. *P*-values were calculated by Kruskal–Wallis tests. **(C, D)** We checked the degree of overlap of age-associated CpG loci between studies by calculating the number of common CpG loci. We performed 100 age-permutation tests with the samples of individual studies to verify the significance of the degree of overlap. Hierarchical clustering results using the degree of overlap of age-associated CpG loci between different studies with different tissue types in normal **(C)** and cancer **(D)**. *P*-value: a Z-test result using the distribution of 10,000 random selections.

Individual studies included samples of various tissue types with different age ranges (Additional file 1: Table S1). Therefore, the average DNAm levels per CpG site were quite different between studies in both disease-free normal samples (*P* < 2.2e–300 using a Kruskal–Wallis test) and cancer samples (*P* < 2.2e–300) (Figure 3A). Similar results were also observed for the average DNAm levels per sample unit (Figure 3B). However, most study pairs with normal samples showed significantly greater degrees of overlap of age-associated CpG loci than would be expected by chance (Figure 3C). Moreover, the results of hierarchical clustering of the *P*-values of the degrees of overlap demonstrated that common age-associated CpG loci were independent of tissue or cell type. In the case of cancer samples, the degrees of overlap of age-associated CpG loci between study pairs were also significant, but less so than for normal samples (Figure 3D).

### Age-associated DNA methylation signatures in normal and cancer

**Figure 4:**
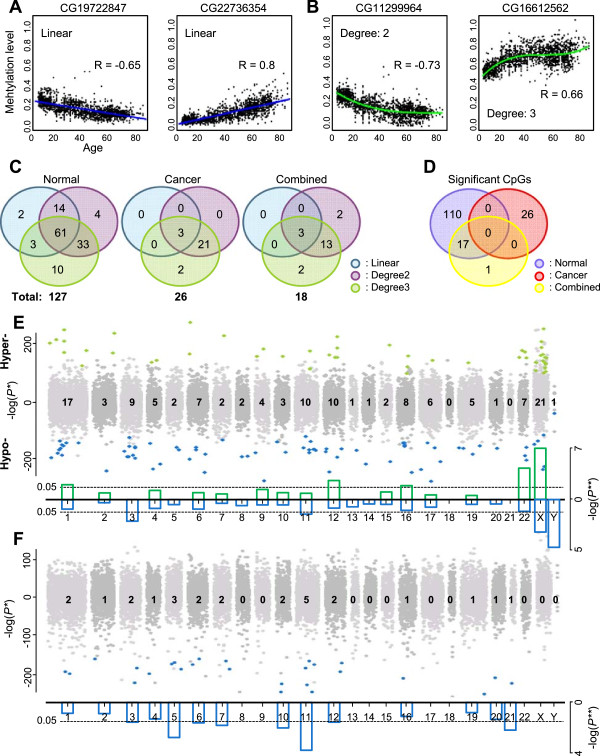
**Age-associated DNA methylation signature independent of tissue type. (A, B)** Examples of age-associated CpG loci with linear **(A)** or nonlinear **(B)** relationships identified in integrated normal samples. **(C)** Venn diagrams showing the numbers of age-associated CpG loci with three regression models (linear and second- and third-degree nonlinear) in integrated normal, cancer, or all samples. **(D)** Venn diagram showing the number of age-associated CpG loci among integrated normal, cancer, and all samples. **(E)** Manhattan plot of age-associated CpG loci in integrated normal samples by chromosome. Hypermethylated CpG loci with age are shown with a –log (*P*-value) and hypomethylated loci are shown with a *log* (*P*-value). The most significant *P*-values among linear and nonlinear models were chosen. Significant loci are marked as green (hypermethylated) or blue (hypomethylated) dots. The numbers of significant age-associated CpG loci by chromosome. Bar plots of *P*-values with hypergeometric tests for the degrees of significance of the numbers of the loci. **(F)** Manhattan plot of age-associated CpG loci in integrated cancer samples by chromosome.

To identify tissue type-invariant age-associated CpG loci, we integrated the normalized DNAm levels of all the disease-free normal samples after removing noisy samples. Using this integrated data set, we first identified CpG loci with a linear relationship with age. For example, the CG19722847 site was linearly hypomethylated (30740381 on chr12; R = -0.65) and CG22736354 was linearly hypermethylated (18230698 on chr6; R = 0.8) according to age, regardless of tissue type (Figure 4A). However, DNAm levels of some loci showed nonlinear patterns according to age (Figure 4B). For these nonlinear relationships, we also observed more rapid changes in DNAm levels at younger ages, which is consistent with previous studies [9, 12]. We also observed similar phenomena for gene-level DNAm levels (Additional file 2: Figure S3A, B). We thus identified tissue type-invariant age-associated DNA methylation signatures using second- and third-degree nonlinear regression models in addition to the linear model. For the threshold, we used three measures, including false discovery rate (FDR) (<0.01), correlation coefficient (≥0.55), and residual error (<0.15), to reduce tissue-type variations. We identified 127 unique CpG loci in the combined normal samples, which we termed a “tissue type-invariant age-associated DNAm signature”. Among these, 80 CpG loci had a linear relationship with age and the other 47 loci were identified using nonlinear models (Additional file 1: Table S2). Seventy-seven loci were hypermethylated and 50 were hypomethylated with age. We also applied a similar approach to examine the DNAm levels of the combined 2181 cancer samples. Compared with normal samples, only 26 age-associated CpG loci were identified (Figure 4C). Interestingly, there was no CpG locus common to the normal and cancer age-associated signatures (Figure 4D). These epigenetic phenomena were also observed with the gene-level DNAm values (Additional file 2: Figure S3C, D). In case of the combined normal and cancer samples, only 18 CpG loci were identified as age-associated. We examined the positions on human chromosomes of the age-associated CpG loci of each of the signatures by separating hypomethylated (blue) and hypermethylated (green) loci, in normal (Figure 4E), cancer (Figure 4F), or combined samples (Additional file 2: Figure S4). Generally, the 127 age-associated loci in normal tissue were distributed throughout the human genome, except for chromosomes 18 and 21. In contrast to a previous study using male pediatric samples [12], the X chromosome had the largest number of age-associated loci. This difference may be caused by differences in sex, age range, and tissue types. We checked the significance of the numbers of loci by chromosome using hypergeometric tests (green bars for hypermethylation and blue bars for hypomethylation with age in Figure 4E). Chromosomes X (*P* = 8.1E–08), 22 (1.3E–03), 12 (1.7E–02), 1 (4.0E–02), and 16 (4.9E–02) were preferentially enriched for hypermethylated loci with age, whereas chromosomes Y (*P* = 3.4E–05), X (9.4E–04), 3 (9.5E–03), and 11 (4.3E–02) were enriched for hypomethylated loci. Thus, the sex chromosomes, especially X, were enriched for age-associated CpG loci in disease-free normal tissues. In cancer samples, chromosomes 3 (*P* = 0.03), 5 (1.7E–03), 6 (0.03), 7 (0.02), 10 (0.01), 11 (1.8E–04), and 21 (0.01) were enriched with age-associated hypomethylated loci (Figure 4F). Interestingly, there were no age-associated loci on the X or Y chromosomes in cancer samples. With the normal and cancer samples matched in age distribution, we also observed similar trends such as no overlap in signature between the normal and cancer samples (Additional file 2: Figure S5).

It was previously suggested that a difference in methylation variation might exist with gender [8]. We therefore identified age-associated signatures for males and females separately (Additional file 2: Figure S6). We found 560 (87 hypermethylated and 473 hypomethylated) and 152 (103 hypermethylated and 49 hypomethylated) CpG loci in the male and female samples, respectively. Even though the number of age-associated loci differed between male and female, their ratio distributions by chromosome were similar (*P* = 0.64 using a Wilcoxon rank-sum test; Additional file 2: Figure S6A). Moreover, the number of hyper- and hypomethylated loci on the X chromosome were similar between males and females (*P* = 0.22 using a Fisher’s test; Additional file 2: Figure S6B).

**Figure 5:**
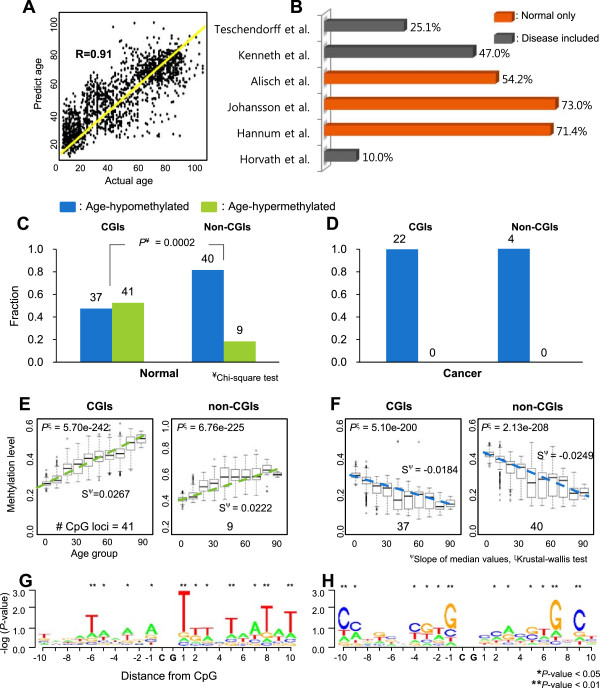
**Characteristics of age-associated DNA methylation signature. (A)** Age prediction using the age-associated normal DNAm signature. Age was predicted with the normal signature using a multivariate linear regression after using a genetic algorithm to identify a feasible set of loci. **(B)** Degrees of overlap with age-associated DNAm signatures identified in previous studies. Overlap percentages were calculated by the common numbers divided by the smaller number of total loci in either study. The studies with only normal samples are orange; other studies including disease samples are gray. **(C, D)** The fractions of hyper- (green) or hypomethylated (blue) CpG loci in the age-associated signatures in normal **(C)** or cancer **(D)** according to genomic regions. The number on each bar indicates the count of the corresponding loci. *P*-value was calculated by a chi-square test. **(E, F)** The hyper- **(E)** or hypomethylation **(F)** patterns according to age group of normal age-associated DNA loci in CGI or non-CGI. Blue or green dotted lines show the linear regressions of median values of individual age groups using only hypo- (blue) or hypermethylated (green) loci, respectively. Numbers below are the counts of loci considered for the corresponding cases. **(G, H)** Nucleotide compositions of the sequences surrounding the hypo- **(G)** or hypermethylated loci **(H)** of normal age-associated DNAm signature. –log (*P*-value) of the y axis was calculated by random selection tests representing overrepresentation for each base at each location of the surrounding CpG.

We built a feasible age-prediction model to see whether the normal 127-site signature could be used as a tissue-invariant age predictor. We applied a multiple linear regression model after identifying a feasible subset of the signature using a genetic algorithm (Methods). The selected age-prediction model was composed of 20 CpG loci of the signature (see “Predicted age” column in Additional file 1: Table S2). The correlation between the actual ages of the combined normal samples and their predicted ages using the model was highly significant (R = 0.91, *P* = 0.002 from 10,000 random selection tests using all loci in the platform; Figure 5A), which indicates that the DNAm levels of the age-associated signature sites can be used to predict the age of tissues, regardless of tissue type. We next compared the age-associated normal signature with those identified in previous studies (Additional file 1: Table S3). Most previous studies identified age-associated loci using a FDR threshold in a linear model. Thus, we compared the loci resulting from only linear regression (FDR < 0.01) and found that 430 age-associated CpG loci were age-associated in the integrated normal samples. For instance, a recent study using the Illumina Human 450 K platform and a linear regression model identified 137993 CpG loci associated with age in blood cells of 421 healthy subjects aged from 14 to 94 years [11]. Of these 137993 loci, the 6696 CpG loci present on the Illumina 27 K overlapped 73% with our 430 age-associated loci. Another study by Day *et al*. [13] found that 4747 CpG loci correlated with age in four tissue types, including brain samples, using a linear regression method, and the degree of overlap with our loci was 47%. Notably, we observed higher degrees of overlap of CpGs with previous studies that used only normal samples than with other studies that included diseased samples (Figure 5B and Additional file 1: Table S3). Sixteen of our 127 age-associated loci were not identified in the previous studies. Interestingly, 13 of these 16 loci were located on the X chromosome (see “Unique CpG” column in Additional file 1: Table S2).

Our collected DNAm profiles included diverse tissue types in the normal samples. We next identified tissue-type-specific age-associated signatures (Additional file 2: Figure S7). Although the number of age-associated loci in normal samples varied from one (for prostate) to 2713 (for peripheral whole blood) loci across tissue types (Additional file 2: Figure S7A), most tissue-type pairs showed significant degrees of overlap of the age-associated CpG loci compared with random expectation, except for the prostate tissue samples (Additional file 2: Figure S7B). In the cancer samples, we could not find significant tissue-type-specific age-associated loci with the threshold we used in most cases.

### Characteristics of tissue type-invariant age-associated DNA methylation signature

We investigated the genomic locations of the loci in the age-associated DNAm signatures in normal or cancer samples. Of the 127 loci in normal samples, 78 were located in CGI regions and the others in non-CGI regions (Additional file 1: Table S4); whereas in cancer, 22 loci were located in CGIs and four in non-CGIs. Thus, while the normal signature was enriched in CGI regions, the cancer signature was even more enriched in CGI regions (Fisher’s exact test, *P* = 0.02). In the normal age-associated signature, hypermethylated loci were enriched in CGI regions (41 age-hypermethylated and 37 age-hypomethylated), whereas hypomethylated loci were enriched in non-CGIs (40 age-hypomethylated and 9 age-hypermethylated) (Chi-square test, *P* = 0.0002; Figure 5C). Interestingly, the cancer signature had only age-hypomethylated loci in both CGI and non-CGI regions (Figure 5D). Similar results were also detected for gene-level DNAm patterns (Additional file 2: Figure S8). We next checked the changing rates of DNA methylation in the normal signature within CGI or non-CGI regions. Age-hypermethylated loci increased more rapidly in CGI regions (Figure 5E). In contrast, hypomethylated loci decreased more rapidly in non-CGI regions (Figure 5F). The cancer signature of only hypomethylated loci showed much smaller changes with age than did the normal signature (Additional file 2: Figure S9).

Next, we investigated the nucleotide composition surrounding the 127 CpG loci in the normal signature (Additional file 2: Figure S10A). The sequences surrounding the normal signature showed significant overrepresentation of thymine (T) residues at -6, -3, +1, +6, +8, and +10 bases from the CpG loci (10,000 random selection tests of all CpG loci in the platform, *P* = 0.031, 0.036, 0.008, 0.036, 0.002, and 0.002, respectively), adenine (A) residues at +3 and +7 bases (*P* = 0.023, 0.035), and guanine (G) residues at -1 base (*P* = 0.04). Interestingly, the sequence motifs surrounding age-associated CpG loci were quite different between hypermethylated and hypomethylated loci. Sequences surrounding age-hypomethylated loci presented AT-rich sequences (Figure 5G), whereas the sequences surrounding age-hypermethylated loci were enriched for GC-rich sequences (Figure 5H); these are also enriched in CGI regions [24]. These phenomena were also observed in the cancer signature (Additional file 2: Figure S10B).

Analysis of gene ontology descriptors for the age-associated DNA methylation signature in normal samples indicated that the aging-related terms such as regulation of protein kinase activity (*P* = 0.01), metabolic processes (*P* = 0.04), immune system processes (*P* = 0.04), and neuron differentiation (*P* = 0.04) were significantly enriched in the CGI regions (Additional file 1: Table S5A). Genes related to age-associated loci in non-CGI regions (*n* = 48) also carried aging-related ontology terms including protein maturation (*P* = 0.04), and negative regulation of cell proliferation (*P* = 0.07) (Additional file 1: Table S5B). In cancer, the aging-related terms such as neuron apoptosis (*P* = 0.02) and muscle organ development (*P* = 0.03) were significantly enriched (Additional file 1: Table S6). We compared the normal signature with bivalent chromatin domain regions. Previously, human aging-associated DNA hypermethylation was found to occur preferentially at bivalent chromatin domains in ES cells [25]. Interestingly, we found that our normal age-associated hypermethylated loci overlapped significantly with the previously reported bivalent regions (*P* = 3.08E–31 using a *Z*-test of 10,000 random selection tests; Additional file 2: Figure S11).

### Disruption of age-associated DNA methylation signature in cancer

**Figure 6:**
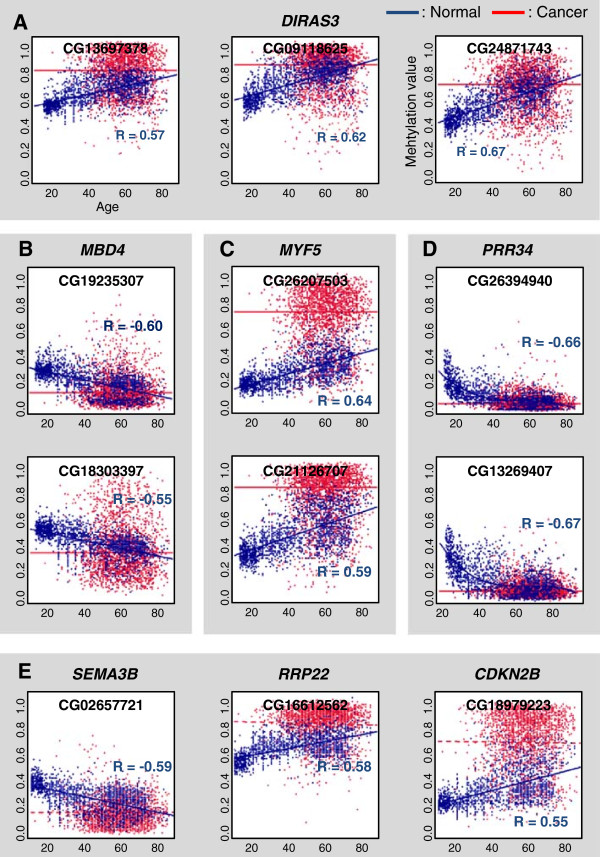
**Disruption of age-associated DNA methylation signature in cancer. (A D)** Plots of DNA methylation patterns of normal age-associated loci affiliated with *DIRAS3* (CG13697378, CG09118625, and CG24871743 in **A)**, *MBD4* (CG19235307 and CG18303397 in **B)**, *MYF5* (CG26207503 and CG21126707 in **C)**, and *PRR34* (CG26394940 and CG13269407 in **D)** in normal (blue) and cancer (red) samples. Blue or red lines are the linear (or nonlinear) regression results for normal or cancer samples, respectively. **(E)** Plots of DNA methylation patterns of tumor suppressor genes including *SEMA3B*, *RRP22* and *CDKN2B*.

For several of the 127 normal age-associated DNAm loci, multiple CpG sites were identified in a single gene. For example, CG13697378 (68285433 on chr1), CG09118625 (68285559 on chr1) and CG24871743 (68285238 on chr1) are located in the DIRAS family, GTP-binding RAS-like 3 (*DIRAS3*) gene. *DIRAS3* is known as a tumor-suppressor gene that is expressed in normal ovarian or breast epithelial cells, but is rarely expressed in tumors [26]. These three loci are in CGI regions that show a positive correlation between DNAm levels and age (R = 0.57, 0.62, or 0.67, respectively) in normal tissues (Figure 6A). In cancer, however, no correlation with age that observed and methylation levels were generally high regardless of age. Interestingly, the age-associated DNAm increases or decreases of the normal signature were aberrantly accelerated in cancer samples, indicating that abnormal acceleration in age-associated DNAm change might induce tumorigenesis. In another example, CG19235307 (130642844 on chr3) and CG18303397 (130642825 on chr3), which are in non-CGI regions, are situated in *MBD4*, methyl-CpG-binding domain protein 4, which is associated with histone-modifying and chromatin-remodeling complexes [27]. These two loci showed a negative correlation between DNAm levels and age in normal tissues. However, the correlation with age disappeared in cancer samples and the DNAm levels of the loci were aberrantly lower (Figure 6B). Similar phenomena were also observed in the multiple age-associated CpG loci in *MYF5* (myogenic factor 5) (Figure 6C) and *PRR34* (Figure 6D).

Of the normal signature, eight genes were common with the tumor suppressor genes and *PTTG1* was common with the proto-oncogenes listed in the UniProt Knowledgebase (http://www.uniprot.org/uniprot/; see “Tumor related” columns in Additional file 1: Table S2) [28]. The degree of overlap between the normal signature and the tumor suppressor genes was significant (*P* = 0.02; hypergeometric test). DNAm levels of some tumor suppressor genes including *RRP22* (CG16612562 located at 28042045 on chr22), *CDKN2B* (CG18979223 located at 21995769 on chr9), and *DIRAS3* increased with age; whereas those of *SEMA3B* (CG02657721 located at 50280835 on chr3) decreased (Figure 6A,E). Those tumor suppressor genes also showed abnormal acceleration in methylation changes in cancer samples.

### Interaction network and sequence conservation analysis of the age-associated DNA methylation signature

**Figure 7:**
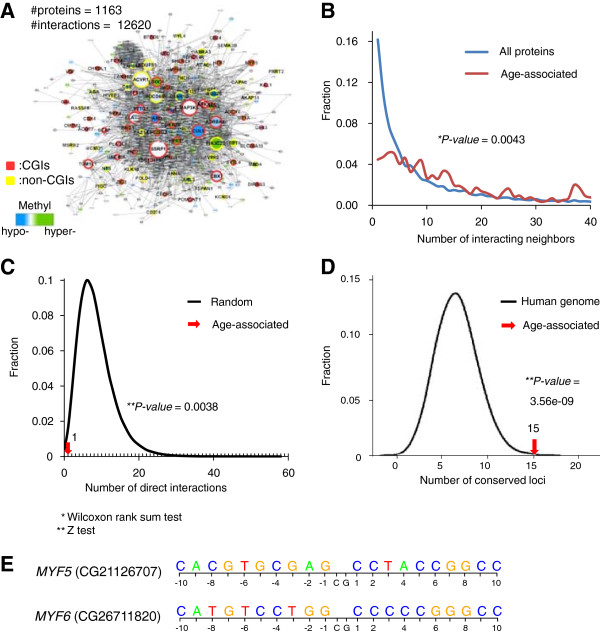
**Network characteristics and sequence conservation of the age-associated DNA methylation signature. (A)** Protein interactions of age-associated DNA methylation genes in normal tissue. Blue or green nodes indicate genes hypo- or hypermethylated with age; gray nodes indicate the interacting neighbors of the age-associated gene products. Red or yellow borders of nodes indicate whether the loci are located in CGIs (red) or non-CGIs (purple). Edges between nodes indicate the protein–protein interactions of gene products. Node sizes are proportional to numbers of interacting proteins. **(B)** The distributions of numbers of interacting protein neighbors from the network shown in **(A)** (in red), or from all protein interactions combined (in blue), respectively. **(C)** The number of direct protein interactions between gene products affiliated with the age-associated DNA methylation signature is indicated by a red arrow. The black curve indicates the distribution of 10^6^ random selections of proteins with the same number of the age-associated genes. **(D)** The number of the age-associated DNA loci with significant the average phyloP scores (>1.3) is indicated by a red arrow. The black curve indicates the background distribution of the human genome. **(E)** Surrounding sequences of age-associated loci in *MYF5* and *MYF6*.

We examined the human protein interaction network of the 127 normal age-associated loci, mapped to 122 unique genes (Figure 7A). For this human protein interaction network analysis, we integrated protein interactions from a number of open databases (Methods). We found that a protein interaction subnetwork that included the first neighbors of 122 gene products under the integrated network included 1163 proteins and 12,620 interactions between them. Analysis of the number of interacting neighbors revealed that the age-associated gene products in the normal signature had relatively more interacting partners than the average for all proteins in the prepared interaction network, and the overall distributions differed significantly between them (Wilcoxon rank-sum test, *P* = 0.0043; Figure 7B). Furthermore, the 122 unique gene products tended not to interact directly with each other: only one interaction existed among the 122 gene products (*Z* test, *P* = 0.0038; Figure 7C). This indicates that the age-associated gene products cover a large portion of the human protein interaction network. We also analyzed the DNA sequence conservation scores of the 127 CpG positions using the average phyloP [29] (Figure 7D). The results showed that 15 CpG positions (“Conserved CpG” in Additional file 1: Table S2) had significantly higher conservation scores (average phyloP score > 1.3) and that this number was significantly higher than random expectations (*P* = 3.56E–09 from a Z test using the phyloP distribution of 10,000 random selection tests). Gene ontology term analysis of the highly conserved loci showed that aging-associated terms such as metabolic processes (*P* = 1.40E–06), muscle system processes (*P* = 8.68E–05), and cell proliferation (*P* = 1.87E–02) were enriched (Additional file 1: Table S7). Of highly conserved loci, *MYF5* and *MYF6* are associated with myogenic regulation, which is related with a decrease of muscle in mass, strength, and contraction in aging [30]. The sequences surrounding *MYF5* and *MYF6* in CGI are enriched guanine and cytosine nucleotides (Figure 7E).

## Conclusion

In this study, we extensively investigated the relation between DNAm and age from a large number of collected samples from a range of diverse studies of normal and cancer tissues. Using these samples and both linear and nonlinear models, we identified tissue type-invariant DNAm age-associated signatures for both healthy normal and cancer samples. We observed that the characteristics of the genomic regions involved in the normal age signature were quite different from those of the cancer signature, and there was no common age-associated locus between them. The normal age signature was particularly enriched on the X chromosome and satisfactorily predicted the chronological ages of samples of many different tissue types. Moreover, the DNAm levels of the normal signature approached the corresponding cancer levels with age. Interaction network analysis showed that normal age-associated gene products had relatively more interacting partners and had a tendency not to interact directly with each other. The genomic sequences of these age-associated loci were also well conserved. The age-associated DNAm increases or decreases of the normal signature were aberrantly accelerated in cancer samples. Although we could not completely address the biological significance of these characteristics, these findings can be used to address important questions in developmental biology and cancer research.

## Methods

### DNA methylation profiles and data processing

We collected human normal or cancer DNA methylation profiles from public databases including GEO (http://www.ncbi.nlm.nih.gov/geo/) and TCGA (http://cancergenome.nih.gov/). We limited the samples to those analyzed with the Illumina Infinium HumanMethylation27 assay to reduce bias between platforms, and excluded studies with fewer than 10 samples and diseases other than cancer. In total, we collected 1812 normal samples and 2181 cancer samples from 13 studies (Additional file 1: Table S1).

To compare and integrate DNA methylation profiles across studies, we downloaded the methylated (M) and unmethylated (U) signal intensities for genomic DNA for each individual study. Normalized DNA methylation levels (β) were calculated as the ratio of signal from a methylated intensity relative to the sum of both methylated and unmethylated intensity; i.e., [M/(M + U)], where β ranges continuously from 0 (unmethylated) to 1 (methylated). For gene-level methylation levels, we averaged the DNAm levels of corresponding loci to individual genes from the Illumina Infinium HumanMethylation27 annotation file.

### Analysis of age-associated DNA methylation signature

Linear regression analysis, with age as the response and DNA methylation as the predictor, was performed separately for each CpG site in each individual study. We also identified tissue type-invariant age-associated signatures using nonlinear regression with second- and third-degree polynomial models. All regression models were fitted using the R function “lm”. The applied FDR correction was calculated using the R function “p.adjust”. Note that in the analysis of integrated samples, we removed noisy samples that had less than 0.5 median correlation coefficients with all other samples. The same methods were applied to the analysis of gene-level methylation patterns, which were mapped using the Illumina annotation files for the HumanMethylation27 platform.

### Random selection and permutation test in the analysis of individual studies

All random selection test and permutation test analyses were conducted in R. Permutation *P*-values for each CpG locus were calculated by assessing the number of significantly age-associated sites for each study under 100 age-permutated data sets (Figure 2E). To check the significance of the degree of overlap between study pairs, we randomly selected the same number of age-associated loci 10,000 times from all the CpG sites in the platform. The *P*-values of the overlaps were calculated from the distributions of the numbers of random overlaps between study pairs (Figure 3C,D).

### Generation of an age-prediction model

The age-prediction model was generated with a multivariate linear model approach based on the genetic algorithm implemented in the R package “genalg” [31]. We identified a suboptimal set of 20 CpG loci from the normal age signature using a genetic algorithm with a conventional linear regression model. The correlation degree in the R package was used to analyze the relationship between predicted and actual ages of samples.

### Analysis of chromosome distribution

Using NCBI Human Genome (build 36.1) and the Illumina annotation files for the HumanMethylation27 platform, CpG loci were mapped to a chromosome. *P*-value significances of the numbers of hypermethylated or hypomethylated loci with age were assessed using hypergeometric tests for each chromosome.

### Analysis of sequence motifs

Sequences of CpG loci from the Illumina annotation files were selected to analyze the 20 base pairs surrounding each CpG dinucleotide in all 27 k probes. We calculated the proportions and degrees of significance of the four types of nucleotides at each base compared with the 10,000 randomly selected sets using all 27 k probes. Logo plots (Figure 5G,H and Additional file 2: Figure S10) were illustrated using the R package “seqLogo” [32].

### Analysis of network and sequence conservation

For human protein–protein interaction networks, we integrated information from well-known open databases, including HPRD [33], BioGRID [34], IntAct [35], MINT [36], Reactome [37] and iRefWeb [38] and the previous result of Lee *et al.*[39]. A total of 136,489 interactions among 14,216 human proteins were prepared. For the network of the normal age signature, we chose the related gene products of the 127 age-associated loci. We also included the proteins that directly interacted with the age-associated gene products, resulting in 12,620 interactions between 1163 human proteins. The network was visualized using Cytoscape [40]. To identify the sequence conservation score of the normal age signature, we downloaded the average phyloP scores from the UCSC Genome Browser [29]. We used the average phyloP scores. Fifteen of the 127 normal age-associated loci had significantly higher conservation scores (phyloP > 1.3). To check the significance of the number of conserved loci, we randomly selected 127 phyloP scores 10,000 times for a background distribution.

### Availability of supporting data

The data sets supporting the results of this article are included within the article and its additional files.

## Electronic supplementary material

Additional file 1: Table S1: DNA methylation data sets used in this study. **Table S2.** Age-associated DNA methylation signature regardless of tissue type. **Table S3.** Characteristics of previous age-associated DNA methylation studies. **Table S4.** Numbers of loci in the age-associated DNAm signatures using the integrated data set. **Table S5.** Top functional annotation clusters of significant differentially aging genes in normal. **Table S6.** Top functional annotation clusters of significant differentially aging genes in cancer. **Table S7.** Top functional annotation clusters of significant differentially aging genes in conseved genes. (PDF 85 KB)

Additional file 2: Figure S1: DNA methylation patterns between normal and cancer samples according to genomic regions. **Figure S2.** Relation with the number of age-associated loci and sample information. **Figure S3.** Gene-level age-associated DNA methylation signature. **Figure S4.** Manhattan plots of age-associated CpG loci in all samples by chromosome. **Figure S5.** (NEXT PAGE) Manhattan plots of age-associated CpG loci in age-matched samples by chromosome. **Figure S6.** Fractions of age-associated CpG loci according to gender. **Figure S7.** Tissue-type-specific age-associated CpG loci. **Figure S8.** The fractions of hyper- or hypomethylated genes in age-associated signatures according to genomic regions. **Figure S9.** The hypomethylation patterns of age-associated DNA loci from cancer samples according to age groups in CGIs (A) or non-CGIs (B). **Figure S10.** Nucleotide composition with surrounding sequences of age-associated DNAm signatures. **Figure S11.** Overlap between bivalent chromatin domain regions and the age-associated hypermethylated loci. (PDF 288 KB)

## Abbreviations

BM: Bone marrow
BRCA: Breast invasive carcinoma
CpG: Cytosine - phosphate - Guanine
CGI: CpG islands
COAD: Colon adenocarcinoma
DNAm: DNA methylation
FDR: False discovery rate
GBM: Glioblastoma multiforme
GEO: Gene expression Omnibus
GO: Gene ontology
KIRC: Kidney renal clear cell carcinoma
OV: Ovarian serous cystadenocarcinoma
PB: Peripheral whole blood
TCGA: The cancer genome Atlas
WB: Whole blood.
