# Supplementary material for: Characterization of age signatures of DNA methylation in normal and cancer tissues from multiple studies
Source: BMC Genomics. 2014 Nov 19;15(1):997. doi: 10.1186/1471-2164-15-997 (PMC4289351; doi:10.1186/1471-2164-15-997)
Supplement: Supplementary file 1 — Additional file 1: Table S1: DNA methylation data sets used in this study. Table S2. Age-associated DNA methylation signature regardless of tissue type. Table S3. Characteristics of previous age-associated DNA methylation studies. Table S4. Numbers of loci in the age-associated DNAm signatures using the integrated data set. Table S5. Top functional annotation clusters of significant differentially aging genes in normal. Table S6. Top functional annotation clusters of significant differentially aging genes in cancer. Table S7. Top functional annotation clusters of significant differentially aging genes in conseved genes. (PDF 85 KB) [file 12864_2014_6827_MOESM1_ESM.pdf]

Table S1 DNA methylation data sets used in this study.

Study	Tissue type	Sample characteristics	Age (years)	Platform	Source
GSE27097	PB	Normal (494)	3-17	Infin.27k	GEO
GSE37008	PB	Normal (99)	12-45	Infin.27k	GEO
GSE19711	PB	Normal (274), Cancer (266)	49-91	Infin.27k	GEO
GSE41037	WB	Normal (394)	16-88	Infin.27k	GEO
GSE32251	BM	Cancer (89)	18-69	Infin.27k	GEO
GSE30760	Uterine cervix	Normal (167), Cancer (48)	19-91	Infin.27k	GEO
GSE32393	Breast	Normal (23), Cancer (114)	20-90	Infin.27k	GEO
GSE26126	Prostate	Normal (86), Cancer (95)	43-73	Infin.27k	GEO
TCGA_GBM	Brain&WB	Cancer (293)	10-89	Infin.27k	TCGA
TCGA_OV	Ovarian&WB	Normal (12), Cancer (574)	26-89	Infin.27k	TCGA
TCGA_BRCA	Breast&WB	Normal (27), Cancer (315)	29-90	Infin.27k	TCGA
TCGA_KIRC	Kidney&WB	Normal (199), Cancer (219)	33-86	Infin.27k	TCGA
TCGA_COAD	Colon&WB	Normal (37), Cancer (168)	36-90	Infin.27k	TCGA

Infin.27k : Illumina HumanMethylation27 BeadChip (HumanMethylation27_270596_v.1.2)
WB : Whole blood, PB : Peripheral whole blood, BM : Bone marrow

Table S2 Age-associated DNA methylation signature regardless of tissue type.

Among linear and second- and third-degree nonlinear regression models, the model with the most significant correlation coefficient was denoted for each CpG locus.

Type	Probe	R	Model	CGI/Non-CGI	Chr	Gene	Predicted age	Unique CpG	Tumor related	Conserved CpG
Normal	CG00047050	-0.56	Degree 2	CGI	11	CUL5				
Normal	CG00169548	-0.63	Degree 2	Non-CGI	14	BAZ1A				
Normal	CG00353953	-0.58	Degree 2	Non-CGI	12	ZNF384				
Normal	CG00398048	-0.64	Degree 3	Non-CGI	4	AGA				
Normal	CG00404599	0.58	Degree 2	CGI	23	TSC22D3				Y
Normal	CG00425710	0.60	Degree 3	CGI	16	MT1A				
Normal	CG00431114	-0.57	Degree 3	Non-CGI	20	C20ORF121				
Normal	CG00582628	-0.63	Degree 3	Non-CGI	8	RGS20				
Normal	CG00657095	-0.55	Degree 2	CGI	6	PPARD	Y			
Normal	CG00962635	0.62	Degree 3	CGI	23	SYTL4		Y		
Normal	CG01135626	0.60	Degree 3	CGI	23	CDX4		Y		
Normal	CG01154193	-0.60	Degree 3	CGI	1	IQCC				
Normal	CG01511567	-0.60	Degree 3	CGI	11	SSRP1				
Normal	CG02228185	-0.65	Degree 3	Non-CGI	17	ASPA				Y
Normal	CG02657721	-0.64	Degree 3	Non-CGI	3	SEMA3B			Tumor suppressor	
Normal	CG02844545	0.56	Linear	CGI	6	GCM2				
Normal	CG03996822	-0.65	Degree 3	Non-CGI	4	RASSF6				
Normal	CG04036898	-0.62	Degree 3	CGI	1	POMGNT1				
Normal	CG04084157	0.66	Degree 3	CGI	7	VGF				
Normal	CG04464446	0.55	Degree 3	CGI	11	GAL				
Normal	CG04474832	-0.55	Degree 3	CGI	3	ABHD14A				
Normal	CG04553232	-0.55	Degree 2	CGI	23	KAL1		Y		
Normal	CG04967538	0.57	Degree 3	CGI	23	CXORF42		Y		
Normal	CG05154390	-0.63	Degree 3	CGI	1	MRPS15				
Normal	CG05294455	0.56	Degree 3	Non-CGI	17	MYL4				Y
Normal	CG05921207	0.56	Degree 3	CGI	23	CHRDL1		Y		
Normal	CG05995267	-0.57	Degree 3	Non-CGI	16	PRRT2				
Normal	CG06291867	0.59	Degree 2	CGI	10	HTR7	Y			Y
Normal	CG06384053	-0.56	Degree 3	Non-CGI	11	FLJ20010				Y
Normal	CG06656924	-0.57	Degree 3	CGI	6	ZNF76				

Normal	CG07388493	-0.57	Degree 3	Non-CGI	1	NDUFS5				
Normal	CG08022502	-0.63	Degree 3	CGI	15	UNC45A				
Normal	CG08090640	-0.72	Degree 3	Non-CGI	17	IFI35				
Normal	CG08209133	0.57	Degree 3	CGI	4	SLC10A4				
Normal	CG08440425	-0.56	Degree 3	CGI	11	LRRC51		Y		
Normal	CG08480367	0.56	Degree 3	CGI	23	SRPX				
Normal	CG08587542	-0.57	Degree 3	Non-CGI	5	KIAA0141				
Normal	CG08587864	-0.55	Degree 3	Non-CGI	1	PIGC				
Normal	CG08750326	-0.69	Degree 3	Non-CGI	23	ODZ1				
Normal	CG08888956	-0.60	Degree 3	Non-CGI	12	NTS	Y			
Normal	CG08996521	-0.58	Degree 3	CGI	3	CISH				
Normal	CG09118625	0.68	Degree 3	CGI	1	DIRAS3	Y		Tumor suppressor	
Normal	CG09155905	-0.56	Degree 3	CGI	17	FNDC8				
Normal	CG09706243	-0.60	Degree 3	Non-CGI	11	POLD4				
Normal	CG10362475	0.59	Degree 3	CGI	11	SHANK2	Y		Tumor suppressor	Y
Normal	CG10453758	-0.55	Degree 3	Non-CGI	3	ACAD11				
Normal	CG10523019	0.60	Degree 3	CGI	2	RHBDD1				
Normal	CG10584819	0.57	Degree 3	CGI	23	HPRT1		Y		
Normal	CG10872209	-0.59	Degree 3	Non-CGI	10	ARL3				
Normal	CG10893437	-0.57	Degree 2	Non-CGI	13	C13ORF8				
Normal	CG11299964	-0.73	Degree 2	CGI	9	MAPKAP1				
Normal	CG11377136	0.57	Degree 3	CGI	22	PKDREJ				
Normal	CG11919694	-0.58	Degree 3	CGI	16	TBC1D10B				Y
Normal	CG12373771	0.55	Linear	CGI	22	CECR6				
Normal	CG12467090	0.56	Degree 3	Non-CGI	1	PIK3C2B				
Normal	CG12688670	-0.57	Degree 3	CGI	16	KIF22				
Normal	CG12883767	0.58	Degree 3	CGI	12	SLC26A10				
Normal	CG12941369	-0.59	Degree 3	Non-CGI	3	PDCD6IP				
Normal	CG13269407	-0.64	Degree 3	CGI	22	PRR34				
Normal	CG13697378	0.61	Degree 3	CGI	1	DIRAS3			Tumor suppressor	
Normal	CG13726191	-0.55	Degree 3	Non-CGI	4	FGFBP1				
Normal	CG13813391	0.59	Degree 3	CGI	16	CMTM2				
Normal	CG14161241	-0.63	Degree 3	CGI	6	PLAGL1		Y	Tumor suppressor	
Normal	CG14377791	-0.60	Degree 3	Non-CGI	19	LOC400696				
Normal	CG14519515	-0.58	Degree 3	CGI	11	ADRBK1				
Normal	CG14562990	0.57	Degree 3	CGI	23	LDOC1		Y		

Normal	CG14654731	0.57	Degree 3	CGI	4	FLJ30834				
Normal	CG15200096	-0.57	Degree 3	Non-CGI	23	KCND1				
Normal	CG15538427	-0.55	Degree 3	Non-CGI	11	LOC221091	Y			
Normal	CG15664701	-0.56	Degree 3	Non-CGI	23	AKAP14		Y		
Normal	CG15776355	-0.60	Degree 3	Non-CGI	12	C1R				
Normal	CG15804973	-0.66	Linear	CGI	6	MAP3K5				
Normal	CG16273597	-0.63	Degree 3	CGI	6	CD83				
Normal	CG16352283	0.65	Degree 3	CGI	1	FAM46B	Y			Y
Normal	CG16612562	0.66	Degree 3	CGI	22	RRP22			Tumor suppressor	
Normal	CG16682903	-0.66	Degree 3	Non-CGI	2	ACVR1				Y
Normal	CG17189778	0.67	Degree 3	Non-CGI	23	AVPR2	Y	Y		
Normal	CG17421623	-0.63	Degree 3	Non-CGI	3	C3ORF9				
Normal	CG17431739	-0.59	Degree 3	Non-CGI	10	MSRB2				
Normal	CG17461214	0.60	Degree 3	Non-CGI	1	CHML				
Normal	CG17861230	0.55	Degree 2	CGI	19	PDE4C	Y			Y
Normal	CG18303397	-0.57	Degree 3	Non-CGI	3	MBD4				
Normal	CG18665563	0.57	Degree 3	CGI	23	HMGB3				
Normal	CG18801806	-0.65	Degree 3	CGI	11	DLNB14				
Normal	CG18979223	0.55	Degree 2	CGI	9	CDKN2B	Y		Tumor suppressor	
Normal	CG19235307	-0.61	Degree 3	Non-CGI	3	MBD4				
Normal	CG19356189	-0.55	Degree 3	Non-CGI	19	KLK10			Tumor suppressor	
Normal	CG19357849	-0.56	Degree 3	CGI	19	ELAVL1				
Normal	CG19395441	-0.56	Degree 2	CGI	12	C12ORF43				
Normal	CG19556572	0.59	Linear	Non-CGI	9	AKNA	Y			
Normal	CG19688503	-0.69	Degree 3	Non-CGI	23	CAPN6				
Normal	CG19713460	0.58	Degree 3	CGI	22	SYNGR1				
Normal	CG19722847	-0.68	Linear	CGI	12	IPO8	Y			
Normal	CG19724470	-0.58	Degree 3	Non-CGI	9	CD274				
Normal	CG19761273	-0.65	Degree 3	Non-CGI	17	CSNK1D				
Normal	CG19945840	0.61	Linear	CGI	1	B3GALT6	Y			
Normal	CG20974196	-0.56	Degree 3	CGI	19	CFD				
Normal	CG21126707	0.60	Degree 3	CGI	12	MYF5	Y			Y
Normal	CG21448423	-0.57	Degree 2	Non-CGI	1	ACOT11	Y			
Normal	CG21501064	0.68	Degree 3	Non-CGI	23	SEPT6		Y		
Normal	CG21655480	0.56	Degree 3	CGI	23	RP11-450P7.3	Y			
Normal	CG21788470	0.57	Degree 2	Non-CGI	12	PTHLH		Y		

Normal	CG22171829	-0.57	Degree 3	CGI	7	PDK4				Y
Normal	CG22736354	0.80	Linear	CGI	6	NHLRC1				Y
Normal	CG22947000	-0.66	Degree 3	Non-CGI	16	BCMO1				
Normal	CG23124451	-0.60	Degree 3	CGI	22	CBX7			Tumor suppressor	
Normal	CG23580000	0.56	Degree 3	CGI	16	ADCY7				
Normal	CG23654549	-0.55	Degree 3	CGI	24	CD24	Y			
Normal	CG23756219	0.65	Degree 3	Non-CGI	23	DRP2		Y		Y
Normal	CG23762517	-0.57	Degree 3	Non-CGI	1	HIVEP3				
Normal	CG24596472	-0.59	Degree 2	CGI	8	TOP1MT	Y			
Normal	CG24768561	0.68	Degree 3	CGI	2	CENTG2				
Normal	CG24871743	0.69	Degree 2	CGI	1	DIRAS3			Tumor suppressor	
Normal	CG24995836	0.60	Degree 3	Non-CGI	23	GABRA3		Y		
Normal	CG25053900	0.56	Degree 3	CGI	23	ARX		Y		
Normal	CG25165880	-0.60	Degree 2	CGI	1	ACOT7				
Normal	CG25410053	0.60	Degree 3	CGI	23	ZIC3				
Normal	CG26207503	0.64	Degree 2	CGI	12	MYF5	Y			
Normal	CG26294850	-0.59	Degree 3	Non-CGI	1	TSPAN1				
Normal	CG26394940	-0.67	Degree 3	CGI	22	PRR34				
Normal	CG26580095	-0.55	Degree 3	CGI	1	BTBD8				
Normal	CG26614073	-0.60	Degree 3	CGI	3	SCAP				
Normal	CG26711820	0.56	Degree 3	CGI	12	MYF6				Y
Normal	CG26775866	-0.57	Degree 3	CGI	5	PTTG1			Proto-oncogene	
Normal	CG27015931	-0.64	Degree 3	CGI	16	MGC50721				
Normal	CG27169020	0.61	Degree 2	CGI	15	BNC1	Y			
Normal	CG27210390	-0.59	Degree 3	CGI	17	TOM1L1				
Tumor	CG00188348	-0.55	Degree 3	CGI	11	BANF1				
Tumor	CG00542846	-0.61	Degree 3	CGI	21	APP				
Tumor	CG03751799	-0.62	Degree 3	CGI	3	SRPRB				
Tumor	CG03859282	-0.63	Degree 3	CGI	16	ANKS3				
Tumor	CG05958659	-0.55	Degree 3	CGI	5	UNQ1912				
Tumor	CG06290168	-0.55	Degree 3	CGI	12	C12ORF24				
Tumor	CG06565684	-0.60	Degree 3	Non-CGI	1	EXTL2			Tumor suppressor	
Tumor	CG06866862	-0.55	Degree 3	CGI	2	ZNF512				
Tumor	CG09435090	-0.59	Degree 3	CGI	4	QDPR				
Tumor	CG11368578	-0.56	Degree 3	CGI	7	CDK5				
Tumor	CG14112945	-0.65	Degree 3	CGI	19	SUV420H2				

Tumor	CG14191360	-0.55	Degree 3	CGI	7	MET	
Tumor	CG14705927	-0.63	Degree 3	CGI	10	P4HA1	
Tumor	CG15302567	-0.67	Degree 3	CGI	11	PPME1	
Tumor	CG17918089	-0.61	Degree 2	CGI	1	SETDB1	
Tumor	CG17995823	-0.59	Degree 3	Non-CGI	11	CTNND1	
Tumor	CG18015044	-0.56	Degree 2	CGI	6	ABCF1	
Tumor	CG18022926	-0.65	Degree 2	Non-CGI	10	MAWBP	
Tumor	CG21829265	-0.56	Degree 2	CGI	6	ZNF451	
Tumor	CG22051763	-0.59	Degree 2	CGI	12	UTP20	
Tumor	CG22176017	-0.63	Degree 2	CGI	11	POLR2G	
Tumor	CG22276571	-0.60	Degree 2	CGI	20	DSTN	
Tumor	CG24146183	-0.56	Degree 2	CGI	11	JMJD2D	
Tumor	CG24341944	-0.57	Degree 2	CGI	3	RSRC1	
Tumor	CG25418748	-0.57	Degree 2	CGI	5	RUFY1	
Tumor	CG25507001	-0.58	Degree 2	Non-CGI	5	NME5	
Combined	CG00582628	-0.60	Degree 2	Non-CGI	8	RGS20	
Combined	CG01154193	-0.59	Degree 2	CGI	1	IQCC	
Combined	CG02657721	-0.57	Degree 2	Non-CGI	3	SEMA3B	Tumor suppressor
Combined	CG03996822	-0.58	Degree 2	Non-CGI	4	RASSF6	
Combined	CG04036898	-0.59	Degree 2	CGI	1	POMGNT1	
Combined	CG08440425	-0.57	Degree 2	CGI	11	LRRC51	
Combined	CG09706243	-0.58	Degree 2	Non-CGI	11	POLD4	
Combined	CG13269407	-0.61	Degree 3	CGI	22	PRR34	
Combined	CG13726191	-0.55	Degree 2	Non-CGI	4	FGFBP1	
Combined	CG14377791	-0.56	Degree 2	Non-CGI	19	LOC400696	
Combined	CG15775914	0.55	Degree 2	Non-CGI	1	CHML	
Combined	CG16612562	0.56	Degree 3	CGI	22	RRP22	Tumor suppressor
Combined	CG17421623	-0.60	Degree 3	Non-CGI	3	C3ORF9	
Combined	CG17461214	0.57	Degree 3	Non-CGI	1	CHML	
Combined	CG19395441	-0.57	Degree 2	CGI	12	C12ORF43	
Combined	CG19556572	0.58	Degree 2	Non-CGI	9	AKNA	
Combined	CG24768561	0.56	Degree 2	CGI	2	CENTG2	
Combined	CG26394940	-0.60	Degree 3	CGI	22	PRR34	

Table S3 Characteristics of previous age-associated DNA methylation studies.

Paper	Platform	Tissue type	# of Sample	Sample characteristics	Method	# of age-associated CpGs	Overlap (%)
Teschendorff et al.	Illumina 27K	WB	261	Healthy wonmen (148)<br>Ovarin cancer (113)	A robust linear regression model (FDR < 0.05)	589	25.1
Alish et al.	Illumina 27K	PB	398	Health pediatric males	A linear fixed-effects regression model (FDR < 0.01)	2078	54.2
Horvath et al.	Illumina 27K	Brain & WB	1688	Healthy (365)<br>Schizophrenia (293)<br>Type1 diabetics (190)<br>Healthy older women (348)<br>Brain (492)	Weighted linear network model	1000 (age-hyper methylation)	10
Johansson et al.	Illumina 450K	White blood cells	421	Healthy (421)	A linear regression model (Bonferroni < 0.05)	137993 (27K : 6696)	73
Hannum et al.	Illumina 450K	WB	482	Healthy (482)	A multivariate linear regression model (FDR < 0.05)	71 (27K : 7)	1.2
Kenneth Day et al.	Illumina 27K	Brain & WB & Kindney & Muscle	283	Normal blood (71)<br>Normal brain (78)<br>Normal kidney (83)<br>Normal muscle (51)	A linear regression model (Bonferroni < 0.05)	4747	47

Table S4 Numbers of loci in the age-associated DNAm signatures using the integrated data set.

(A) Numbers of CpG-level signatures.

CpG loci	CGIs	non-CGIs	Total	*P*-value*
Normal	78	49	127	0.02
Cancer	22	4	26	
Combined	8	10	18	

(B) Numbers of gene-level signatures.

Genes	CGIs	non-CGIs	Total	*P*-value*
Normal	31	33	64	0.17
Cancer	12	5	17	
Combined	2	1	3	

*Fisher's exact test

Table S5 Top functional annotation clusters of significant differentially aging genes in normal.

(A) Enriched GO terms of genes corresponding to 78 CpGs in CGI regions.

GO terms	Count	Genes	*P* -value	Fold enrichmnet
Regulation of protein kinase activity	6	MAP3K5, ADCY7, DIRAS3, CDKN2B, CD24, CISH	0.01	4.44
Regulation of kinase activity	6	MAP3K5, ADCY7, DIRAS3, CDKN2B, CD24, CISH	0.01	4.29
Negative regulation of cell proliferation	6	PPARD, CUL5, CDKN2B, CD24, LDOC1, GAL	0.01	4.24
Regulation of transferase activity	6	MAP3K5, ADCY7, DIRAS3, CDKN2B, CD24, CISH	0.01	4.12
Negative regulation of lymphocyte activation	3	HMGB3, CD24, GAL	0.02	14.18
Regulation of lymphocyte activation	4	CD83, HMGB3, CD24, GAL	0.02	6.9
Response to organic substance	8	CD83, ADCY7, CDKN2B, ADRBK1, CD24, HPRT1, GAL, VGF	0.02	2.83
Negative regulation of leukocyte activation	3	HMGB3, CD24, GAL	0.02	13.43
Regulation of transcription from RNA polymerase II promoter	8	PLAGL1, MYF6, PPARD, CDX4, ZNF76, MYF5, ZIC3, SCAP	0.02	2.81
Negative regulation of cell activation	3	HMGB3, CD24, GAL	0.02	12.55
Regulation of lymphocyte differentiation	3	CD83, HMGB3, CD24	0.02	12.15
Regulation of leukocyte activation	4	CD83, HMGB3, CD24, GAL	0.03	6.15
Regulation of cell activation	4	CD83, HMGB3, CD24, GAL	0.03	5.83
Regulation of cell proliferation	8	ARX, PPARD, CUL5, CDKN2B, BNC1, CD24, LDOC1, GAL	0.03	2.59
Regulation of phosphorylation	6	MAP3K5, ADCY7, DIRAS3, CDKN2B, CD24, CISH	0.03	3.29
Regulation of phosphorus metabolic process	6	MAP3K5, ADCY7, DIRAS3, CDKN2B, CD24, CISH	0.04	3.16
Regulation of phosphate metabolic process	6	MAP3K5, ADCY7, DIRAS3, CDKN2B, CD24, CISH	0.04	3.16
Negative regulation of immune system process	3	HMGB3, CD24, GAL	0.04	9.23
Cerebral cortex neuron differentiation	2	ARX, HPRT1	0.04	46.41

(B) Enriched GO terms of genes corresponding to 49 CpGs in non-CGI regions.

GO terms	Count	Genes	*P* -value	Fold enrichmnet
Protein maturation	3	PTHLH, AGA, C1R	0.04	44.67
Negative regulation of cell proliferation	4	PTHLH, CD274, ODZ1, FGFBP1	0.07	4.05

Table S6 Top functional annotation clusters of significant differentially aging genes in cancer.

Enriched GO terms of genes corresponding to 22 CpGs in CGI regions.

GO terms	Count	Genes	*P* -value	Fold enrichmnet
Adult behavior	3	APP, MET, CDK5	0	27.76
Cell motion	4	APP, MET, CDK5, DSTN	0.02	6.7
Neuron apoptosis	2	APP, CDK5	0.02	83.76
Muscle organ development	3	APP, MET, CDK5	0.03	11.31
Endocytosis	3	APP, RUFY1, CDK5	0.03	10.85
Membrane invagination	3	APP, RUFY1, CDK5	0.03	10.85
Visual learning	2	APP, CDK5	0.03	58.95
Visual behavior	2	APP, CDK5	0.04	51.34
Dendrite development	2	APP, CDK5	0.04	45.47
Protein amino acid phosphorylation	4	APP, RSRC1, MET, CDK5	0.04	4.77

Table S7 Top functional annotation clusters of significant differentially aging genes in conseved genes.

Enriched GO terms of genes and those first neighbors corresponding to 15 genes.

GO terms	Count	Genes	*P* -value
Regulation of activin receptor signaling pathway	5	ACVR1, INHBA, ACVR1B, ACVR2B, ACVR2A	2.98E-12
Positive regulation of multicellular organismal process	7	ACVR1, HDAC2, MYC, INHBB, INHBA, ACVR2B, ACVR2A	4.00E-08
Regulation of cell cycle	8	HDAC2, JUN, CREBBP, CSNK2A1, HDAC1, MYC, CSNK2A2, INHBA	4.77E-08
Positive regulation of myeloid cell differentiation	4	JUN, INHBA, ACVR1B, ACVR2A	1.43E-07
Positive regulation of developmental process	7	ACVR1, JUN, MYC, INHBA, ACVR1B, ACVR2B, ACVR2A	1.82E-07
Muscle contraction	3	IKBKB, CALM3, CALM1	6.71E-05
Muscle system process	3	IKBKB, CALM3, CALM1	8.68E-05
Positive regulation of cellular metabolic process	9	ACVR1, HDAC2, JUN, CREBBP, HDAC1, MYC, INHBA, ACVR1B, ACVR2A	1.40E-06
Growth	4	INHBB, INHBA, ACVR2B, INHBC	1.42E-04
Regulation of immune system process	4	JUN, INHBA, ACVR1B, ACVR2A	2.07E-03
Skeletal muscle contraction	1	IKBKB	7.25E-03
Cell proliferation	3	CREBBP, MYC, ACVR2A	1.87E-02
