# Supplementary material for: Characterization of age signatures of DNA methylation in normal and cancer tissues from multiple studies
Source: BMC Genomics. 2014 Nov 19;15(1):997. doi: 10.1186/1471-2164-15-997 (PMC4289351; doi:10.1186/1471-2164-15-997)

Figure S1 DNA methylation patterns between normal and cancer samples according to genomic regions. Box plots for the average methylation levels of normal (Normal) or cancer (Cancer) samples according to genomic regions (CGIs and non-CGIs) for individual studies. P-values were calculated by Wilcoxon rank-sum tests.

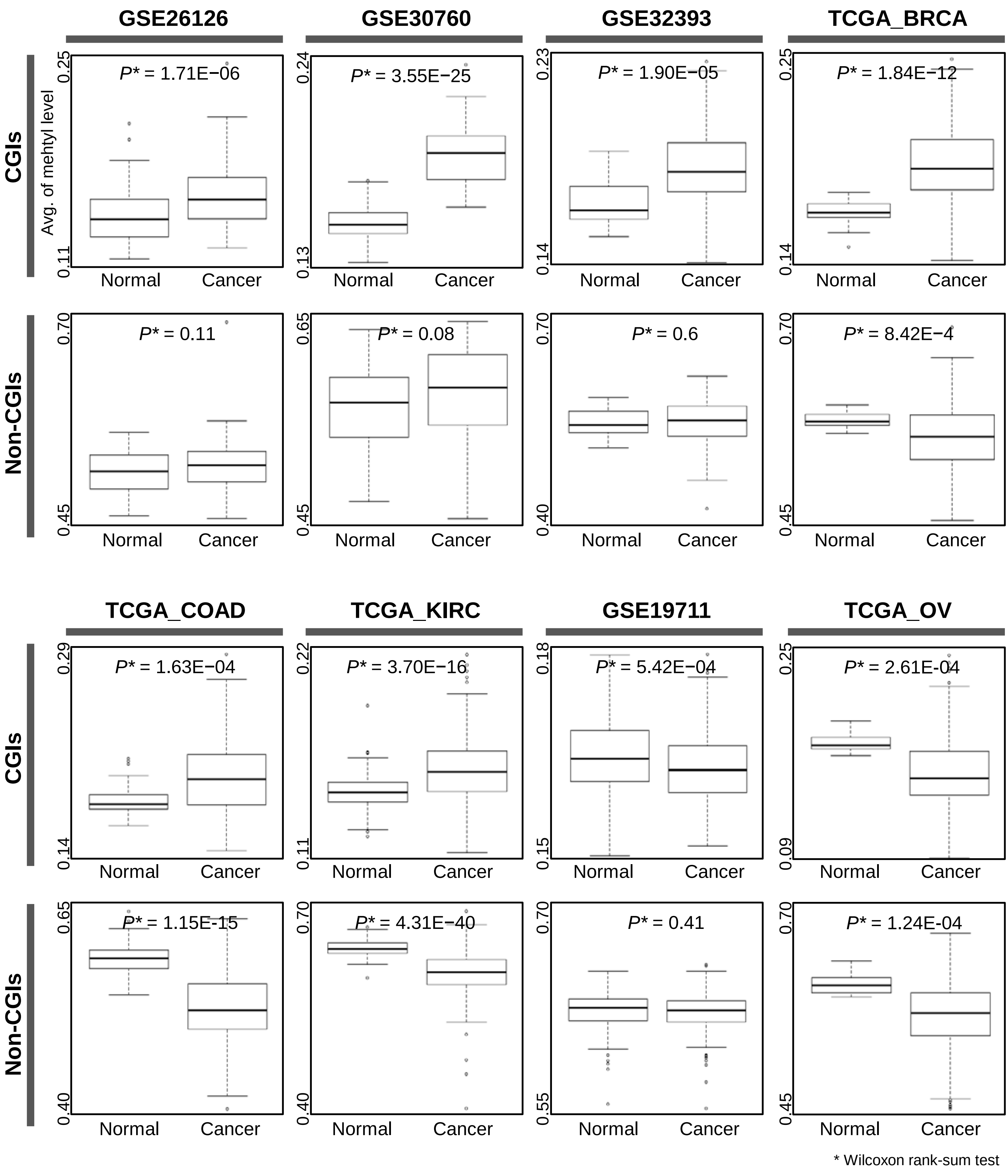

Figure S2 Relation with the number of age-associated loci and sample information. (A, B) Each point is a study corresponding to the count of significant loci and the sample number in normal (A) or cancer (B). **(C, D)** Significant counts and age ranges of samples in individual studies in normal (C) or cancer (D). Many age-associated loci were observed in the GSE41037 study because of the wide age range (16–88 years) and in the GSE30760 study because of the large number of samples (n = 167). For the GSE26126 study, however, a relatively small number of loci were identified as age-associated because of the narrow age range (43–73), even though it included a large number of samples (n = 86).

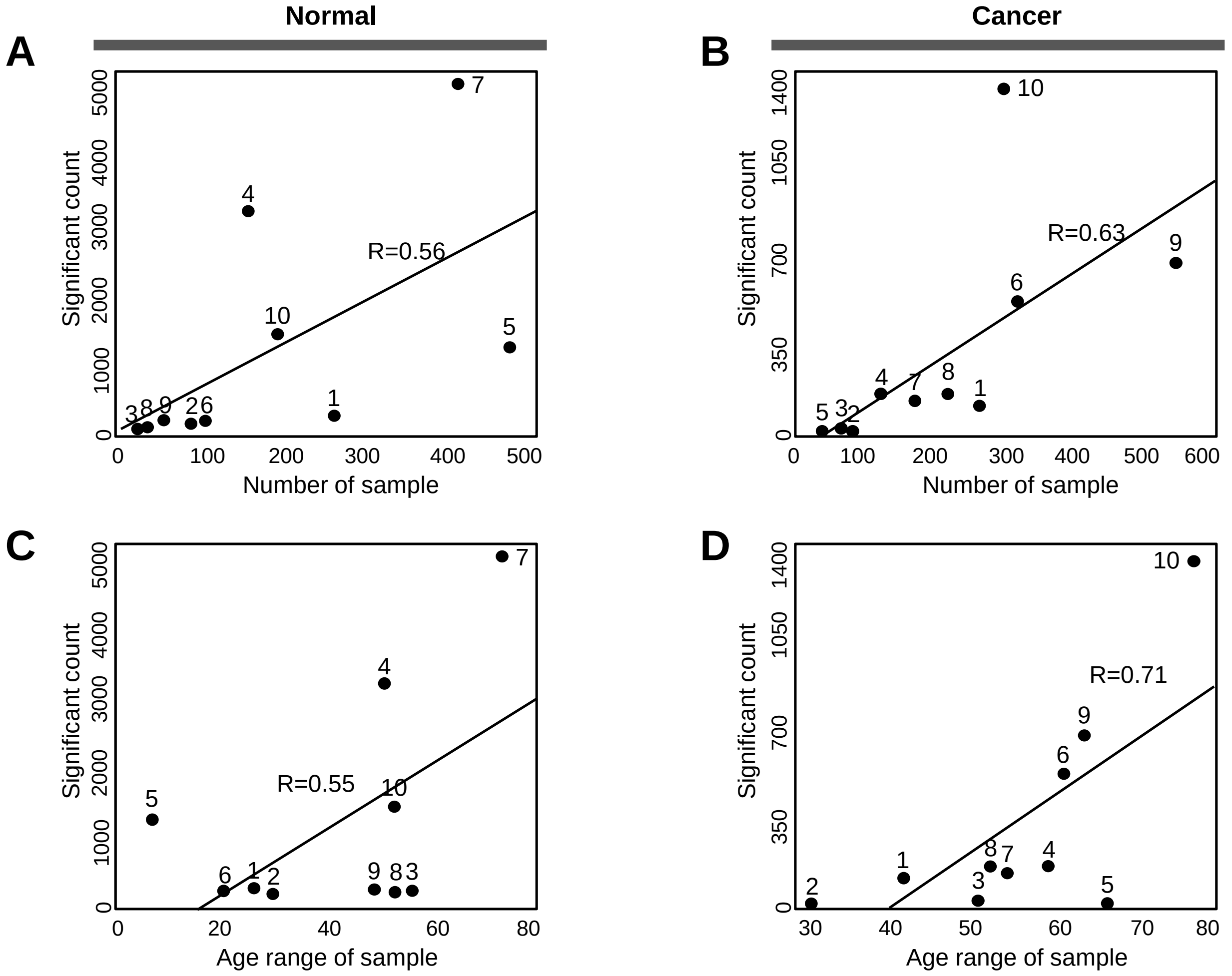

1: GSE19711, 2: GSE26126, 3: GSE32393, 4: GSE30760, 5: GSE27097, 6: GSE37008, 7: GSE41037, 8: TCGA_BRCA, 9:TCGA_COAD, 10: TCGA_KIRC (in **A** and **C**).

1: GSE19711, 2: GSE26126, 3: GSE32251, 4:GSE32393, 5:GSE30760, 6: TCGA_BRCA, 7: TCGA_COAD, 8: TCGA_KIRC, 9: TCGA_OV, 10: TCGA_GBM (in **B** and **D**).

Figure S3 Gene-level age-associated DNA methylation signature. (A, B) Examples of age-associated DNA methylation genes with a linear (A) or nonlinear (B) relationship. Gene-level methylation was calculated from the average of the affiliated DNA loci. **(C)** Venn diagrams showing the numbers of age-associated genes in three regression models (linear and second- and third-degree nonlinear) in integrated normal, cancer, or all samples. **(D)** Venn diagram showing the number of age-associated genes in integrated normal, cancer, and all samples.

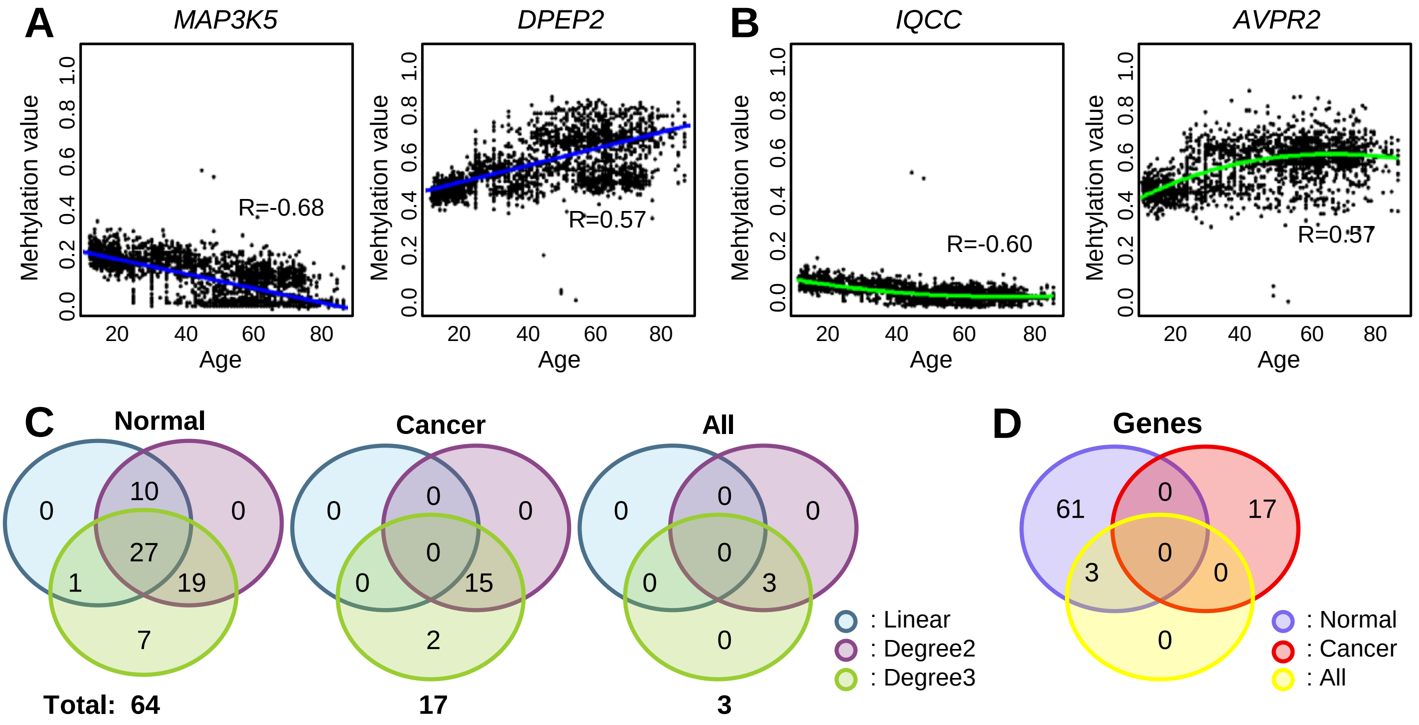

Figure S4 Manhattan plots of age-associated CpG loci in all samples by chromosome. Hypermethylated CpG loci with age are shown with a –log(*P*-value) and hypomethylated loci are shown with a log(*P*-value). The most significant *P*-values among linear and nonlinear models were chosen. Significant loci are marked as green (hypermethylated) or blue (hypomethylated) dots. The numbers of significant age-associated CpG loci by chromosome. Bar plots of *P*-values with hypergeometric tests.

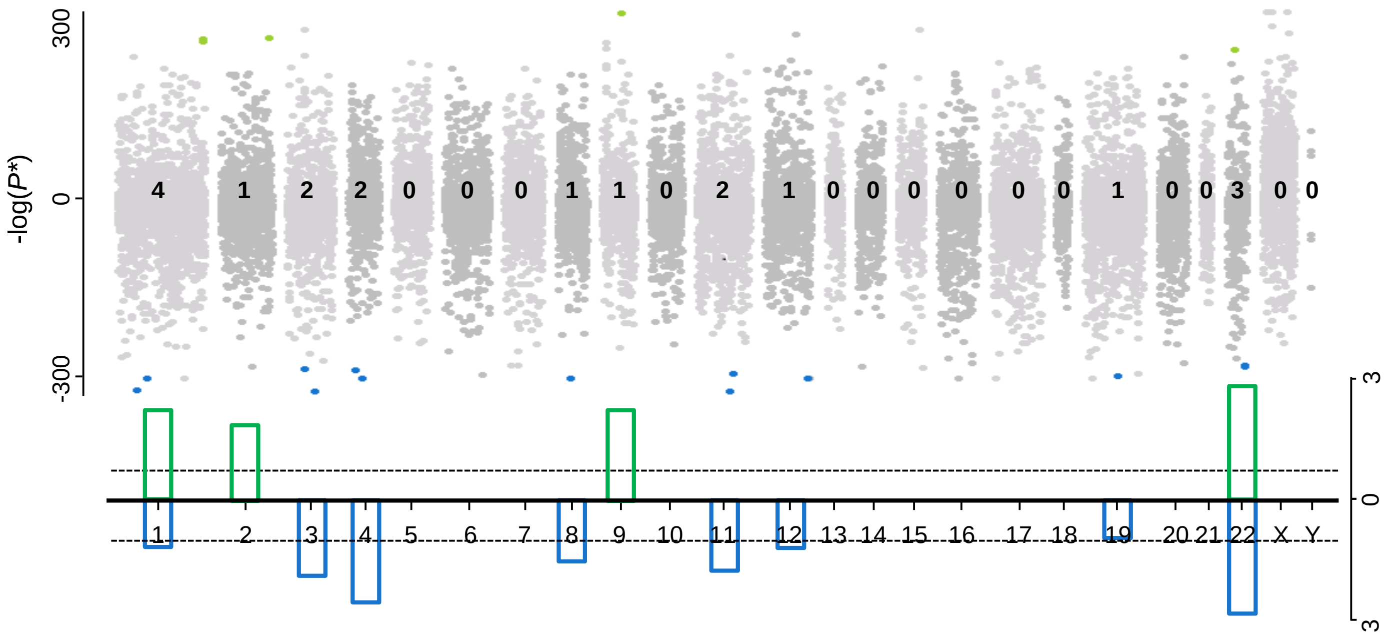

Figure S5 (NEXT PAGE) Manhattan plots of age-associated CpG loci in age-matched samples by chromosome. (A) To reduce the bias from the age difference, we matched the age ranges between normal and cancer samples. We only included normal samples in the age range of cancer since the normal age range is wider than cancer. We identified 29 age-associated CpG loci in normal. **(B, C)** In addition, we exactly matched the age distributions between normal and cancer samples by random sampling with smaller numbers of samples between normal and cancer according to age groups. Here, we used the threshold of $R > 0.32$ and identified new 444 (B) and 61 (C) loci in normal and cancer as age-associated, respectively. Hypermethylated CpG loci with age are shown with a –log(*P*-value) and hypomethylated loci are shown with a log(*P*-value). The most significant *P*-values among linear and nonlinear models were chosen. Significant loci are marked as green (hypermethylated) or blue (hypomethylated) dots. The numbers of significant age-associated CpG loci by chromosome are depicted. Bar plots of *P*-values with hypergeometric tests.

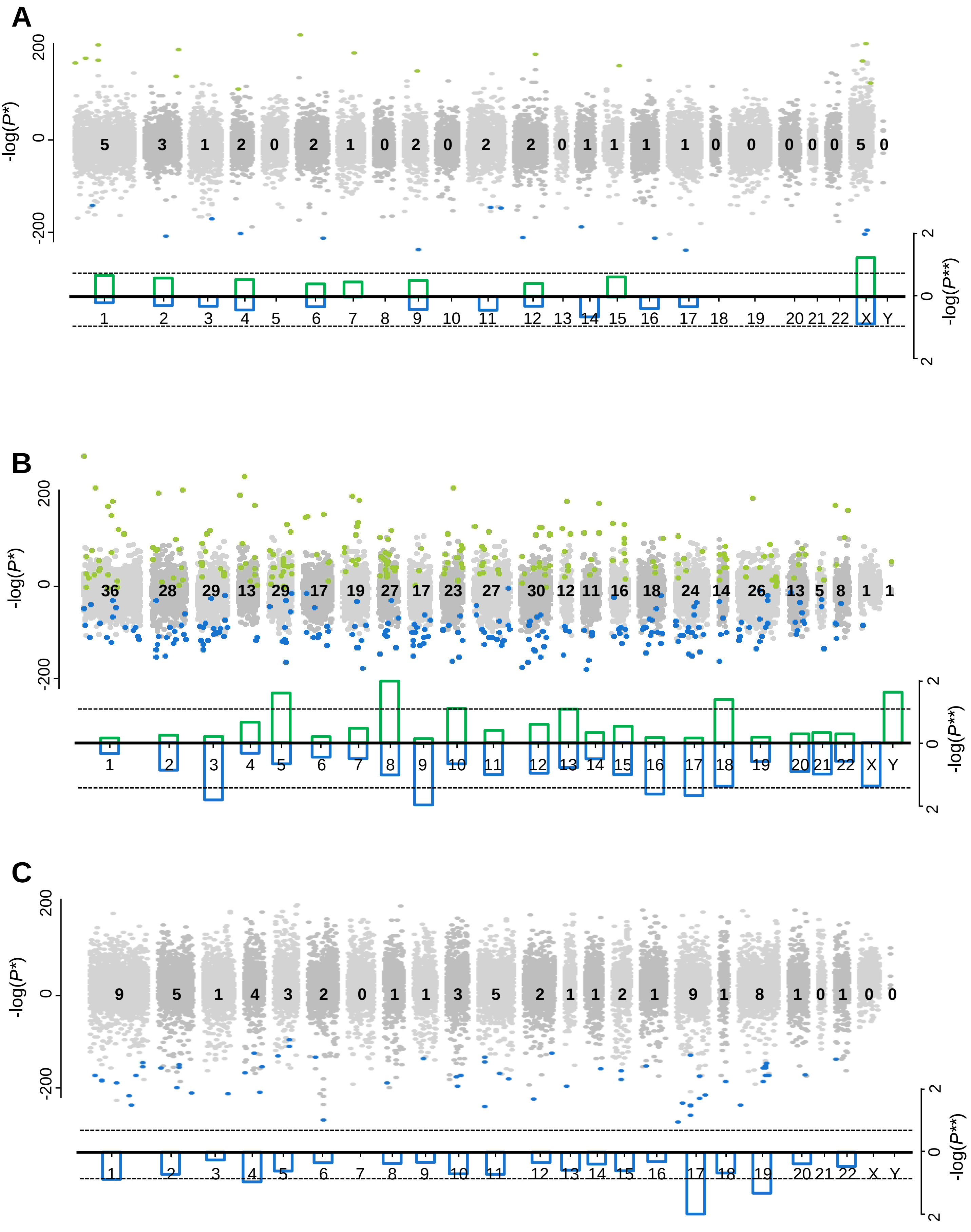
A
-log(P*)
200
0
-200
5 3 1 2 0 2 1 0 2 0 2 2 0 1 1 1 1 0 0 0 0 0 5 0
-log(P**)
2
0
2
1 2 3 4 5 6 7 8 9 10 11 12 13 14 15 16 17 18 19 20 21 22 X Y
B
-log(P*)
200
0
-200
36 28 29 13 29 17 19 27 17 23 27 30 12 11 16 18 24 14 26 13 5 8 1 1
-log(P**)
2
0
2
1 2 3 4 5 6 7 8 9 10 11 12 13 14 15 16 17 18 19 20 21 22 X Y
C
-log(P*)
200
0
-200
9 5 1 4 3 2 0 1 1 3 5 2 1 1 2 1 9 1 8 1 0 1 0 0
-log(P**)
2
0
2
1 2 3 4 5 6 7 8 9 10 11 12 13 14 15 16 17 18 19 20 21 22 X Y

Figure S6 Fractions of age-associated CpG loci according to gender. (A) Bar plot for the fractions of age-associated loci by chromosome in normal male (as dark blue) or female (as pink) samples. **(B)** The numbers of hyper- or hypomethylated loci on **X** chromosome with gender. *P*-value was calculated by a Fisher's exact test.

A

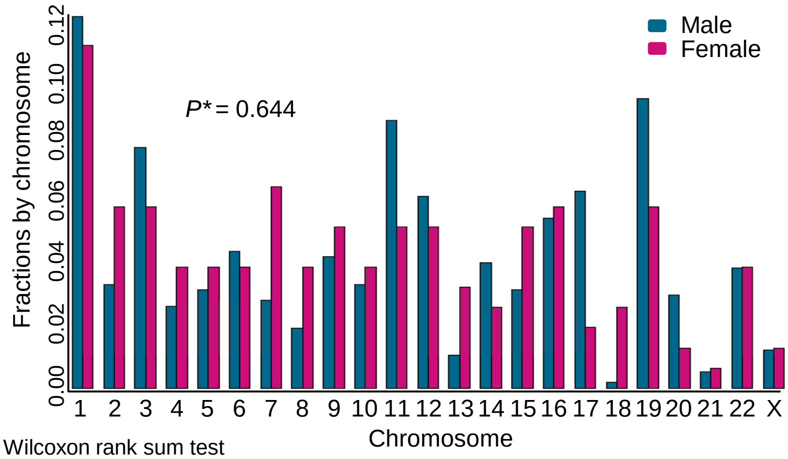

* Wilcoxon rank sum test

B

CpG loci on X chr	Hyper	Hypo	Total	*P*-value*
Male	0	7	7	0.222
Female	1	1	2	

* Fisher's exact test

Figure S7 Tissue-type-specific age-associated CpG loci. (A) The numbers of significant age-associated CpG loci according to distinct types of normal tissue. Numbers in parentheses indicate the counts of samples. **(B)** Hierarchical clustering results using the overlap degrees of age-associated CpG loci between different tissue types in normal. *P*-value: a Z-test result using the distribution of 10,000 random selections.

A

Tissue type	Normal
PB	**2713 (867)**
WB	**115 (394)**
Kidney	**36 (199)**
Cervix	**194 (167)**
Breast	**53 (50)**
Prostate	**1 (86)**
Colon	**17 (37)**

NA: Not Available
WB : Whole blood
PB : Peripheral whole blood
BM : Bone marrow

B

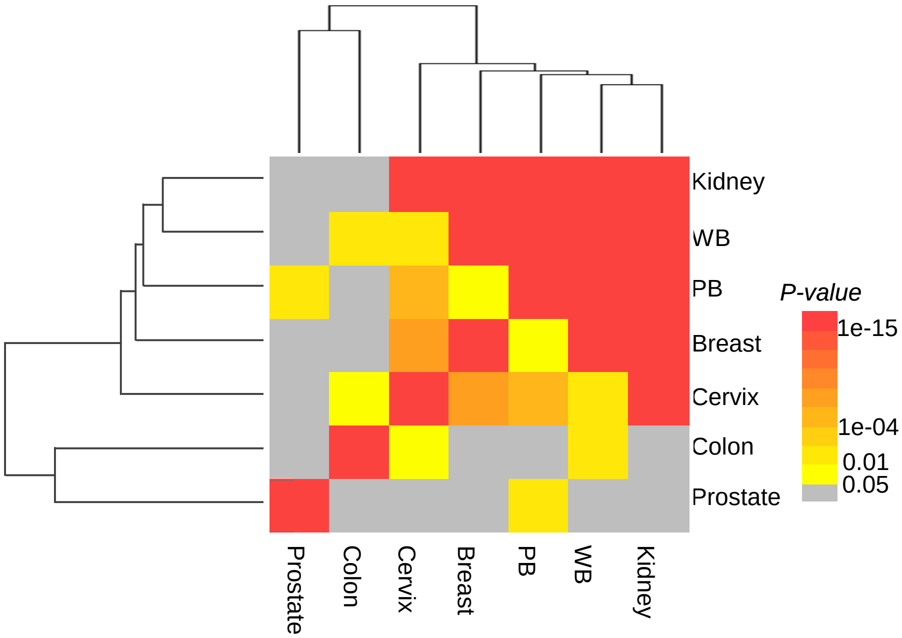

Figure S8 The fractions of hyper- or hypomethylated genes in age-associated signatures according to genomic regions. (A, B) The fractions of hyper- (green) or hypomethylated (blue) genes in the age-associated signatures in normal (A) or cancer (B) tissues, respectively. The number on each bar indicates the count of corresponding genes. *P*-values were calculated by a chi-square test.

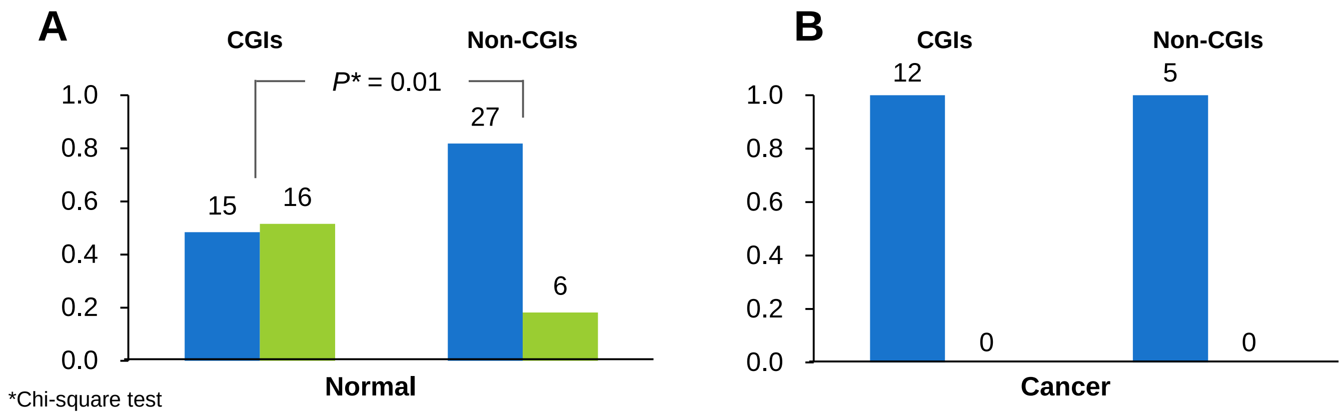

Figure S9 The hypomethylation patterns of age-associated DNA loci from cancer samples according to age groups in CGIs (A) or non-CGIs (B). The blue dotted lines are the linear regressions of median values of individual age groups using only hypomethylated loci.

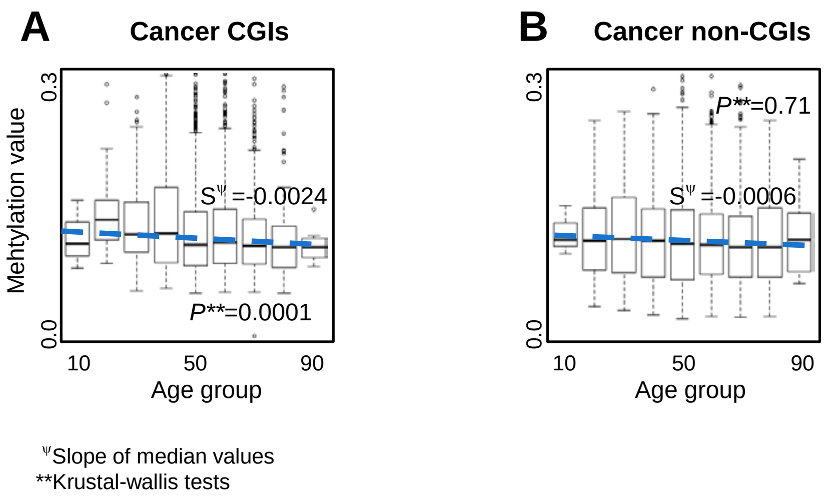

Figure S10 Nucleotide composition with surrounding sequences of age-associated DNAm signatures. – log(*P*-value) of the y axis was calculated by a permutation test representing overrepresentation for each base at each location of the surrounding CpG loci in the 127 normal age-associated signature **(A)** and the 26 cancer signature **(B)**.

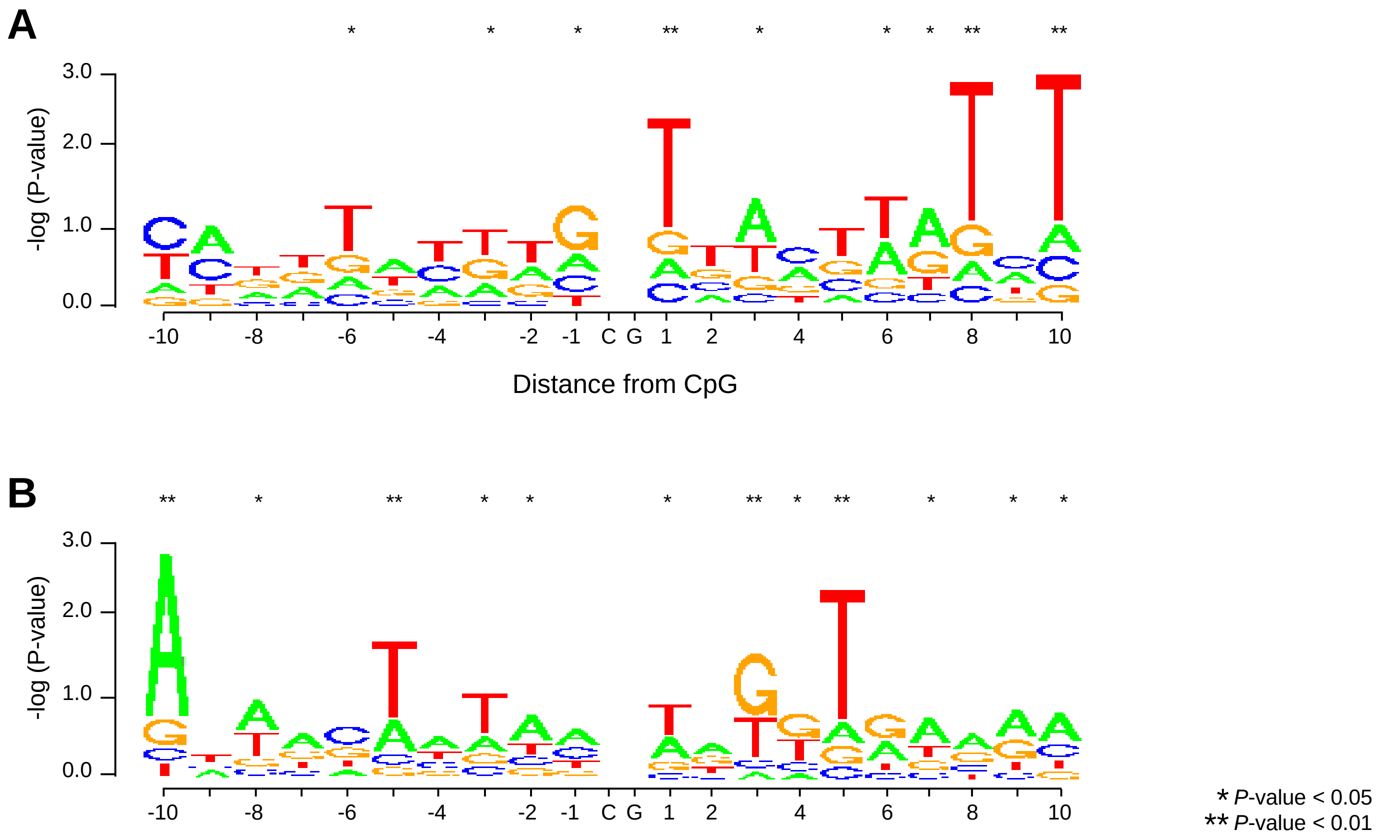

Figure S11 Overlap between bivalent chromatin domain regions and the age-associated hypermethylated loci. The overlap degree with the age-associated hypermethylated loci and the bivalent regions is indicated by a red arrow. The black curve indicates the background distribution of 10000 random selections on the human genome.

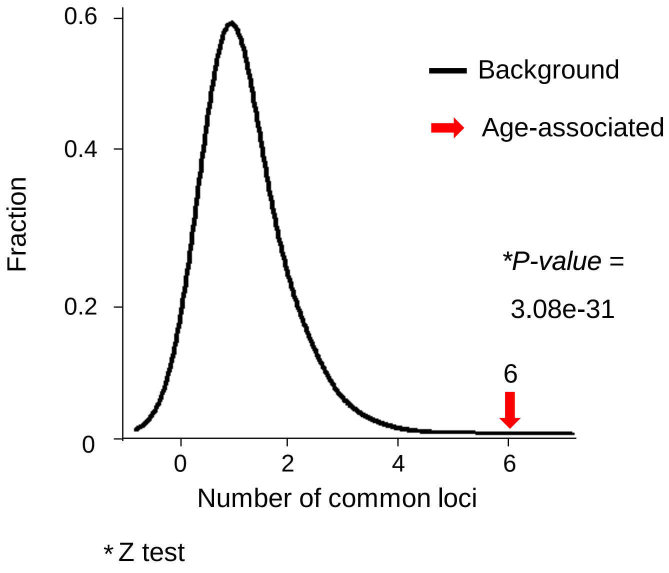

Supplement: Supplementary file 2 — Additional file 2: Figure S1: DNA methylation patterns between normal and cancer samples according to genomic regions. Figure S2. Relation with the number of age-associated loci and sample information. Figure S3. Gene-level age-associated DNA methylation signature. Figure S4. Manhattan plots of age-associated CpG loci in all samples by chromosome. Figure S5. (NEXT PAGE) Manhattan plots of age-associated CpG loci in age-matched samples by chromosome. Figure S6. Fractions of age-associated CpG loci according to gender. Figure S7. Tissue-type-specific age-associated CpG loci. Figure S8. The fractions of hyper- or hypomethylated genes in age-associated signatures according to genomic regions. Figure S9. The hypomethylation patterns of age-associated DNA loci from cancer samples according to age groups in CGIs (A) or non-CGIs (B). Figure S10. Nucleotide composition with surrounding sequences of age-associated DNAm signatures. Figure S11. Overlap between bivalent chromatin domain regions and the age-associated hypermethylated loci. (PDF 288 KB) [file 12864_2014_6827_MOESM2_ESM.pdf]
